# Using Bayesian Evidence Synthesis Methods to Incorporate Real-World Evidence in Surrogate Endpoint Evaluation

**DOI:** 10.1177/0272989X231162852

**Published:** 2023-03-30

**Authors:** Lorna Wheaton, Anastasios Papanikos, Anne Thomas, Sylwia Bujkiewicz

**Affiliations:** Biostatistics Research Group, Department of Health Sciences, University of Leicester, UK; Biostatistics Research Group, Department of Health Sciences, University of Leicester, UK; GlaxoSmithKline R&D Centre, GlaxoSmithKline, Stevenage, UK; Leicester Cancer Research Centre, University of Leicester, Leicester, UK; Biostatistics Research Group, Department of Health Sciences, University of Leicester, UK

**Keywords:** surrogate endpoint, bivariate meta-analysis, real-world evidence

## Abstract

**Objective:**

Traditionally, validation of surrogate endpoints has been carried out using randomized controlled trial (RCT) data. However, RCT data may be too limited to validate surrogate endpoints. In this article, we sought to improve the validation of surrogate endpoints with the inclusion of real-world evidence (RWE).

**Methods:**

We use data from comparative RWE (cRWE) and single-arm RWE (sRWE) to supplement RCT evidence for the evaluation of progression-free survival (PFS) as a surrogate endpoint to overall survival (OS) in metastatic colorectal cancer (mCRC). Treatment effect estimates from RCTs, cRWE, and matched sRWE, comparing antiangiogenic treatments with chemotherapy, were used to inform surrogacy patterns and predictions of the treatment effect on OS from the treatment effect on PFS.

**Results:**

Seven RCTs, 4 cRWE studies, and 2 matched sRWE studies were identified. The addition of RWE to RCTs reduced the uncertainty around the estimates of the parameters for the surrogate relationship. The addition of RWE to RCTs also improved the accuracy and precision of predictions of the treatment effect on OS obtained using data on the observed effect on PFS.

**Conclusion:**

The addition of RWE to RCT data improved the precision of the parameters describing the surrogate relationship between treatment effects on PFS and OS and the predicted clinical benefit of antiangiogenic therapies in mCRC.

**Highlights:**

- Regulatory agencies increasingly rely on surrogate endpoints when making licensing decisions, and for the decisions to be robust, surrogate endpoints need to be validated. In the era of precision medicine, when surrogacy patterns may depend on the drug’s mechanism of action and trials of targeted therapies may be small, data from randomized controlled trials may be limited.
- Real-world evidence (RWE) is increasingly used at different stages of the drug development process. When used to enhance the evidence base for surrogate endpoint evaluation, RWE can improve inferences about the strength of surrogate relationships and the precision of predicted treatment effect on the final clinical outcome based on the observed effect on the surrogate endpoint in a new trial.
- Careful selection of RWE is needed to reduce risk of bias.

Surrogate endpoints are often used when it takes too long, is too expensive, or is too difficult to observe treatment effects on the final clinical outcome of interest.^1^ However, before surrogate endpoints can be used, for example, for regulatory approvals, they should be validated.^2,3^ Surrogate endpoints can be validated based on 3 levels of association: 1) biological plausibility, 2) individual-level surrogacy, and 3) trial-level surrogacy.^4^ However, identifying and validating potential surrogate endpoints can be difficult when data for such analysis are limited. Traditionally, surrogate endpoint evaluation has been carried out using only data from randomized controlled trials (RCTs). Shortages of RCT data are becoming more common as precision medicine evolves and treatments become more effective and are targeted to specific patient populations, leading to smaller cohorts of patients, where it takes longer to observe the treatment effect on the final outcome with reasonable precision.^5–7^ This is due to fewer events recorded in patients receiving targeted therapies and thus high uncertainty around the effectiveness estimates and, as a consequence, around the estimates of association between the treatment effects on the surrogate endpoint and final outcome. It is therefore possible that a putative surrogate endpoint cannot be validated, and treatments may not be granted conditional approval based on treatment effects on the questionable surrogate endpoint, or, if regulatory approval is granted based on an unreliable surrogate endpoint, approval may be withdrawn at the re-evaluation stage when more data on the final outcome are collected, resulting in a waste of resources.^2^ However, in recent years, there has been increased interest in the use of real-world evidence (RWE) at all stages of drug development.^8–16^ While RWE is subject to a higher risk of bias compared with data from RCTs, RWE also has many advantages. It has the potential to increase the evidence base for decision making, often includes data recorded over longer follow-up times, and can be more generalizable to the target population.^17–19^ The addition of RWE could improve validation of surrogate endpoints that could not be validated using RCT data alone.

In this article, we explored how RWE can be used to strengthen the evidence base for surrogate endpoint evaluation. We made use of comparative RWE (cRWE) and single-arm RWE (sRWE) to supplement RCT data on the effectiveness of antiangiogenic therapies in metastatic colorectal cancer (mCRC). We then investigated the impact of the addition of RWE on the estimates of the surrogate relationship between treatment effects on progression-free survival (PFS) and overall survival (OS).

The remainder of this article is structured as follows. Data sources and the statistical methods are described in the “Methods” section. The results are presented in the next section, which is followed by discussion and conclusions in the fourth and fifth sections, respectively.

## Methods

### Data Sources

#### RCTs

Data were obtained from a prior literature review conducted by Ciani et al.,^20^ which included treatment effect estimates from 11 RCTs in mCRC that assessed antiangiogenic treatments such as bevacizumab, combined with various chemotherapies, such as FOLFOX. In the review, Ciani et al.,^20^ defined OS as time from randomization to time of death and PFS as time from randomization to tumor progression or death from any cause.

For this article, we extracted the treatment effects (logHRs) on PFS and OS. RCTs were included in the analysis only when the control arm was chemotherapy, and the treatment arm was an antiangiogenic treatment plus chemotherapy.

#### Comparative real-world evidence

We carried out a literature review to identify cRWE evaluating antiangiogenic treatments for mCRC. The following combinations of terms were used to search for studies published between 2000 and 2020 in the PubMed database: 1) “metastatic colorectal cancer”; 2) “cohort,”“cohort study,”“retrospective,” or “prospective”; 3) “PFS”; 4) “OS”; and 5) “antiangiogenic” or “bevacizumab.”

Abstracts, titles, and, where necessary, full articles were screened, and studies that were not relevant were removed. LogHRs on PFS and OS and their corresponding standard errors were extracted from the remaining studies. To account for potential bias, cRWE studies were included only if they reported treatment effects adjusted for baseline characteristics or potential confounders.

#### Single-arm real-world evidence

Papanikos^21^ identified 16 single-arm observational studies evaluating antiangiogenic treatments alone. We carried out a literature review of single-arm studies of chemotherapy alone, which were subsequently used as control arms. The following combinations of terms were used to search for single-arm studies on chemotherapy published between 2000 and 2020 in the PubMed database: 1) “metastatic colorectal cancer”; 2) “chemotherapy”; 3) “cohort,”“cohort study,”“retrospective,” or “prospective”; 4) “progression” or “PFS”; and 5) “overall survival” or “OS.”

The following terms could not be contained in the title or abstract of the studies: “antiangiogenic,”“bevacizumab,”“cetuximab,”“aflibercept,”“randomised trial,”“randomized trial,” or “phase.” These terms were excluded to prevent RCTs and cRWE being returned. Any additional studies found outside the database search were also included.

### Matching Single-Arm Studies

Unlike RCTs and cRWE, treatment effects cannot be extracted from sRWE as single-arm studies do not make comparisons. To obtain relative treatment effects in the absence of individual participant data (IPD), sRWE studies were matched using aggregate-level data according to the method proposed by Schmitz et al.^22^ The distance $\Delta_{tot}$ between any 2 single-arm studies j and k was determined as the weighted average of differences in covariates.

(1) $$\Delta_{tot}[j,k]=\frac{\sum_{c=1}^{n}w_{c}\cdot \Delta_{c}[j,k]}{\sum_{c=1}^{n}w_{c}}$$

where n is the number of covariates, $w_{c}$ refers to the covariate weights, and $\Delta_{c}[j,k]$ is the difference in covariate c , between studies j and k , scaled to ensure its values are between 0 and 1. The 0 to 1 scaling is applied to ensure all covariates have the same impact on distance before applying the weights. The covariate weights were decided based on rankings from a consensus statement.^23^ This distance takes a value between 0 and 1 , where smaller values indicate more similar studies. Distance measures between treatment arms for RCTs and cRWE were also calculated. There is no consensus on how small the distance measure should be for 2 single-arm studies to be considered sufficiently similar and thus suitable for matching. Therefore, the maximum distance measure observed between arms of RCTs was selected as the maximum allowable distance for matching single-arm studies. The maximum distance measure observed between arms in cRWE studies was selected as the maximum allowable distance for matching sRWE studies in a sensitivity analysis. Pairs of single-arm studies achieving a distance measure below the matching threshold were considered for matching, while pairs of single-arm studies above the matching threshold were not matched. Each single-arm study could be included only once (only in a single matched pair) to avoid double counting of data. Where multiple matches were possible, matches with the smallest distance measure were used.

### Obtaining Treatment Effects for Matched Single-Arm Studies

WebPlotDigitizer was used to extract data from Kaplan–Meier curves from each arm of the matched sRWE studies. Kaplan–Meier curves from RCTs and cRWE were also digitized to compare digitized and reported logHRs. Data from risk tables, reporting the number of patients at risk in each arm at regular time intervals, was also extracted to improve approximated IPD.^24^ Where risk tables were not reported, the number of patients and total number of events in each arm were used.

Data extracted from Kaplan–Meier curves and risk tables were used in Stata to reconstruct IPD using the **ipdfc** command by Wei and Royston.^25^ The Cox proportional hazards model with a single covariate for treatment arm was used to analyze the reconstructed IPD to obtain logHRs on PFS and OS for matched sRWE studies, cRWE, and RCTs.

### Meta-analytic Methods for Surrogate Endpoint Evaluation

Before candidate surrogate endpoints are used in evaluation of a new health technology, they should be validated, by evaluating the strength of the association pattern between the treatment effects on the surrogate and final clinical outcomes and by assessing their ability to predict treatment effects on the final outcome, given treatment effects on the surrogate endpoint.^26^

The standard model for surrogate endpoint evaluation by Daniels and Hughes,^27^ denoted here as D&H, and bivariate random-effects meta-analysis (BRMA) using the product normal formulation (PNF) were used as alternative methods to model the correlated treatment effects (logHRs) on the surrogate endpoint (PFS) and final outcome (OS) using a Bayesian framework. The models were applied to RCT data alone, RCTs and cRWE, and RCTs, cRWE, and matched sRWE. Sensitivity analyses to vague prior distributions were conducted for both models.

#### D&H model

Daniels and Hughes proposed that the observed treatment effects measured on the surrogate endpoint $\left(Y_{1i}\right)$ and final outcome $\left(Y_{2i}\right)$ come from a bivariate normal distribution and estimate the underlying true effects on the surrogate and final outcomes ( $\delta_{1i}$ and $\delta_{2i}$ ) from each study i with corresponding within-study standard deviations ( $\sigma_{1i}$ and $\sigma_{2i}$ ) and within-study correlation $\left(\rho_{\mathrm{wi}}\right)$ :

(2) $$\left(\begin{array}{c} Y_{1i}\\ Y_{2i} \end{array}\right)\sim N\left(\left(\begin{array}{c} \delta_{1i}\\ \delta_{2i} \end{array}\right),\left(\begin{array}{cc} \sigma_{1i}^{2} & \sigma_{1i}\sigma_{2i}\rho_{\mathrm{wi}}\\ \sigma_{1i}\sigma_{2i}\rho_{\mathrm{wi}} & \sigma_{2i}^{2} \end{array}\right)\right)$$

The true effects measured on the surrogate endpoint $\left(\delta_{1i}\right)$ are assumed to be independent in each study. It is also assumed there is a linear relationship between the true treatment effects on the final outcome and the surrogate endpoint, where the intercept $\left(\lambda_{0}\right)$ , slope $\left(\lambda_{1}\right)$ , and conditional variance $\left(\psi_{2}^{2}\right)$ are used as criteria for evaluating the surrogate relationship.

(3) $$\delta_{2i}|\delta_{1i}\sim N\left(\lambda_{0}+\lambda_{1}\delta_{1i},\psi_{2}^{2}\right)$$

Daniels and Hughes considered a surrogate relationship perfect when the following conditions were met: (a) $\lambda_{0}=0$ , (b) $\lambda_{1}\neq 0$ , and (c) $\psi_{2}^{2}=0$ . These conditions state that 1) no treatment effect on the surrogate endpoint implies no treatment effect on the final outcome; 2) the slope is not zero, implying an association between treatment effects on the surrogate and final outcomes; and 3) treatment effects on the final outcome can be perfectly predicted by treatment effects on the surrogate endpoint. The 3 criteria set out by Daniels and Hughes $\left(\lambda_{0}=0,\;\lambda_{1}\neq 0,\;\psi_{2}^{2}=0\right)$ were used to evaluate the surrogate relationship between treatment effects on PFS and treatment effects on OS in mCRC when using the D&H model.

To implement this model in a Bayesian framework, noninformative prior distributions were placed on the fixed effects $\delta_{1i}\sim N\left({0,10}^{4}\right)$ and regression parameters $\lambda_{0,1}\sim N\left({0,10}^{4}\right)$ . To ensure a vague prior distribution on the conditional variance, a uniform prior distribution was placed on the conditional standard deviation $\psi_{2}\sim \mathrm{Unif}\left(0,2\right)$ . A sensitivity analysis was conducted using a prior distribution of $\psi_{2}\sim \mathrm{Unif}\left(0,100\right)$ . A minimally informative prior distribution $\rho_{\mathrm{wi}}\sim \mathrm{Unif}\left(0,1\right)$ was placed on the within-study correlation. In a prior publication, Papanikos^21^ obtained IPD from 1 RCT^28^ included in this analysis and used bootstrapping to obtain a within-study correlation of 0.52. To assess the robustness of the model, a sensitivity analysis was conducted assuming a within-study correlation of 0.52 for all studies.

#### BRMA (PNF)

The D&H model does not estimate correlation or study-level $R^{2}$ , which are often used to assess the strength of a surrogate relationship.^29–31^ For example, the German Institute for Quality and Efficiency in Healthcare defined an acceptable surrogate endpoint by setting a lower bound for the confidence interval (CI) on the correlation coefficient to be 0.85.^32^ To estimate the between-studies correlation and study-level $R^{2}$ , we used BRMA PNF. The most popular form of the BRMA was described by van Houwelingen et al.^33^ and Riley et al.,^34^ while Bujkiewicz et al.^35^ proposed a parameterization of this model such that the between-studies model could be presented as a product of univariate conditional distributions in the so-called PNF. BRMA PNF has the same within-study model as the D&H model (2), but the between-studies model assumes exchangeability of the correlated true (random) treatment effects on both outcomes. In the PNF, the bivariate normal distribution is represented as a sequence of univariate conditional distributions:

(4) $$\left\{ \begin{array}{c} \delta_{1i}\sim N\left(\eta_{1},\psi_{1}^{2}\right)\\ \delta_{2i}|\delta_{1i}\sim N\left(\eta_{2i},\psi_{2}^{2}\right)\\ \eta_{2i}=\lambda_{0}+\lambda_{1}\delta_{1i} \end{array}\right.$$

where $\delta_{1i}$ and $\delta_{2i}$ are the true effects in the population, which are correlated, assumed exchangeable, and normally distributed.

The parameters of the BRMA PNF model can be represented in terms of the parameters of the bivariate normal distribution using the following formulae.^36^

(5) $$\psi_{1}^{2}=\tau_{1}^{2},\psi_{2}^{2}=\tau_{2}^{2}-\lambda_{1}^{2}\tau_{1}^{2},\lambda_{1}=\frac{\tau_{2}}{\tau_{1}}\rho ,$$

where $\tau_{1}$ and $\tau_{2}$ are the between-studies heterogeneity parameters and ρ is the between-studies correlation. To implement this model in a Bayesian framework, vague prior distributions were placed on the between-studies parameters $\tau_{1,2}\sim \mathrm{Unif}\left(0,2\right)$ , ρ~Unif(−1,1) and the intercept $\lambda_{0}\sim N\left({0,10}^{4}\right)$ , implying prior distributions on $\lambda_{1}$ , $\psi_{1}^{2}$ , and $\psi_{2}^{2}$ through rearranging the relationships (5). To assess the robustness of the model, sensitivity analyses were conducted assuming a within-study correlation of 0.52 for all studies and prior distributions of $\tau_{1,2}\sim \mathrm{Unif}\left(0,100\right)$ on the between-studies heterogeneity parameters.

In addition to the surrogacy criteria from the D&H model, a perfect surrogate relationship is defined in the BRMA PNF model when ρ=±1 .^26^ This implies a perfect linear association between treatment effects on the surrogate endpoint and final outcome. The study-level $R^{2}$ in this random-effects model is equal to $\rho^{2}$ .^31^ The 3 criteria set out by Daniels and Hughes ( $\lambda_{0}=0$ , $\lambda_{1}\neq 0$ , $\psi_{2}^{2}=0$ ) and ρ=±1 were used to evaluate the surrogate relationship between treatment effects on PFS and treatment effects on OS in mCRC when using the BRMA PNF model. In addition, we report the between-study correlation with values of ρ=±1 corresponding to a perfect surrogate relationship.

#### Accounting for bias

Non-randomized studies are susceptible to additional risk of bias due to lack of randomization and unmeasured confounding, and this additional bias can manifest in different treatment effects between different study designs.^37^ To account for potential systematic differences in treatment effects between RCTs, cRWE, and sRWE, the BRMA PNF model was extended in the following way. The between-studies model (4) remains the same for all studies, and the within-study model (2) remains the same for RCTs. However, the within-study models for cRWE and matched sRWE include bias terms, $\alpha_{\mathrm{ji}}$ and $\beta_{\mathrm{ji}}$ , respectively, for the surrogate and final outcomes ( j=1,2 ), where the bias terms can differ for each individual RWE study (i):

(6) $$\left(\begin{array}{c} Y_{1i}\\ Y_{2i} \end{array}\right)\sim N\left(\left(\begin{array}{c} \delta_{1i}+\alpha_{1i}\\ \delta_{2i}+\alpha_{2i} \end{array}\right),\left(\begin{array}{cc} \sigma_{1i}^{2} & \sigma_{1i}\sigma_{2i}\rho_{\mathrm{wi}}\\ \sigma_{1i}\sigma_{2i}\rho_{\mathrm{wi}} & \sigma_{2i}^{2} \end{array}\right)\right)$$

(7) $$\left(\begin{array}{c} Y_{1i}\\ Y_{2i} \end{array}\right)\sim N\left(\left(\begin{array}{c} \delta_{1i}+\beta_{1i}\\ \delta_{2i}+\beta_{2i} \end{array}\right),\left(\begin{array}{cc} \sigma_{1i}^{2} & \sigma_{1i}\sigma_{2i}\rho_{\mathrm{wi}}\\ \sigma_{1i}\sigma_{2i}\rho_{\mathrm{wi}} & \sigma_{2i}^{2} \end{array}\right)\right)$$

The bias terms for cRWE and sRWE for each endpoint ( j=1,2 ) for each study are assumed to come from a single normal distribution with a mean and variance:

(8) $$\alpha_{1i}\sim N\left(\alpha_{1},\sigma_{\alpha_{1}}^{2}\right)$$

(9) $$\alpha_{2i}\sim N\left(\alpha_{2},\sigma_{\alpha_{2}}^{2}\right)$$

(10) $$\beta_{1i}\sim N\left(\beta_{1},\sigma_{\beta_{1}}^{2}\right)$$

(11) $$\beta_{2i}\sim N\left(\beta_{2},\sigma_{\beta_{2}}^{2}\right)$$

Additional non-informative prior distributions were placed on the bias terms, $\alpha_{1,2}\sim N\left({0,10}^{4}\right)$ and $\beta_{1,2}\sim N\left({0,10}^{4}\right)$ .

#### Cross-validation

The bivariate nature of the D&H and BRMA PNF models allow both validation of a surrogate endpoint and prediction of an unobserved treatment effect on the final outcome given observed treatment effects on the surrogate endpoint.

To assess the predictive value of a candidate surrogate endpoint, a “take-one-out” cross-validation procedure was conducted for the D&H and BRMA PNF models. The cross-validation was carried out with RCT data alone, RCTs and cRWE, and RCTs, cRWE, and matched sRWE. The cross-validation uses a take-one-out procedure, which is repeated as many times as the number of studies in the data set. For example, when conducting cross-validation on RCTs alone and the number of RCTs in the data set is given by $n_{\mathrm{RCT}}$ , the cross-validation procedure will be repeated $N=n_{\mathrm{RCT}}$ times.

For each study i(i=1,...,N) , the treatment effect on the final outcome, $Y_{2i}$ , was removed and assumed missing at random. The treatment effect on the final outcome was predicted from the treatment effect on the surrogate endpoint, conditional on data on both outcomes from all other studies in the meta-analysis. The mean predicted effect is equal to the mean predicted true effect from Markov chain Monte Carlo (MCMC) simulation. The variance of the predicted effect is $\sigma_{2i}^{2}+\mathrm{var}({\hat{\delta }}_{2i}|Y_{1i},\sigma_{1i},Y_{1(-i)},Y_{2(-i)})$ , where $Y_{1,2(-i)}$ are the observed treatment effects on the surrogate and final outcomes for the remaining studies not omitted in the ith iteration.^3,27^ For a valid surrogate, we expect the 95% predicted interval to contain the true treatment effect in 95% of studies. However, the true treatment effects are unknown in the cross-validation, which limits the cross-validation to comparing the predicted treatment effect estimates with the observed (but assumed missing) treatment effects on the final outcome.

To assess the accuracy of predictions on the final outcome, we compare the predicted treatment effect on OS $\left({\hat{Y}}_{2j}\right)$ to the observed treatment effect on OS $\left(Y_{2j}\right)$ by summarizing the absolute discrepancy between these values. For a perfectly predicted study, the absolute discrepancy will be zero. If the absolute discrepancy decreased with the addition of RWE, this would indicate that the addition of RWE improves the accuracy of predictions. To assess the precision of predictions on the final outcome, we compare the width of the 95% predicted interval $\left(w_{{\hat{Y}}_{2j}}\right)$ and the width of the 95% observed CI $\left(w_{Y_{2j}}\right)$ by summarizing the ratio of these 2 values $\left(w_{{\hat{Y}}_{2j}})/(w_{Y_{2j}}\right)$ . If the ratio of the widths decreased with the addition of RWE, this would indicate that addition of RWE improves precision of predictions.

### Software and Computing

All models were implemented using WinBUGS,^38^ in which estimates were obtained using MCMC simulation with 150,000 iterations (including 50,000 burn-in). Convergence was checked via visual assessment of history, density, and autocorrelation plots. Posterior estimates are presented as means (for approximately normal posterior) or medians (for skewed posterior) with 95% credible intervals (CrI). R was used for data manipulation, to execute WinBUGS code using the R2WinBUGS package,^39^ and to produce figures using the ggplot2 package.

## Results

### Summary of Data

Of the 11 RCTs obtained from the prior literature review, 4 were excluded for not investigating the effect of antiangiogenics in combination with chemotherapy against chemotherapy alone. Overall, 7 RCTs were included in the analysis. Details of these studies can be seen in Table A1 in Appendix A.

The database search of PubMed returned 166 publications for cRWE studies and 145 publications for sRWE studies on the chemotherapy arm. After screening titles, abstracts, and, where appropriate, full articles, 7 cRWE studies comparing bevacizumab against chemotherapy remained, and 8 sRWE studies of chemotherapy alone remained. Of the 7 cRWE studies, 4 adjusted for covariates. However, none of the 4 cRWE studies adjusted for all of the covariates recommended in the consensus statement, and thus treatment effects from cRWE could still be subject to considerable bias. Details of the covariates adjusted for are available in Table B1 in Appendix B.

The consensus statement identified 14 characteristics to include in the recommended set of baseline characteristics and a further 22 characteristics to include in the suggested set of baseline characteristics. Details of these baseline characteristics can be found in Table C1 in Appendix C. However, only 5 covariates were reported by all sRWE studies, and of these, sex was the only covariate not included in the recommended set of baseline characteristics. Following the consensus statement ranking, sex was given weight 1 and all other covariates weight 2. The covariates selected for matching were treatment line (weight = 2, current mean treatment line scaled assuming range 1–3), age (weight = 2, median age scaled assuming range 18–100), performance score (weight = 2, mean Eastern Cooperative Oncology Group/World Health Organization score scaled assuming range 0–3), tumor location (weight = 2, proportion with colon tumor compared with rectum tumor), and sex (weight = 1, proportion of females). An example of calculating the distance measure is available in Appendix D.

Table 1 shows the distance measures between the sRWE studies. A maximum distance measure of 0.030 was applied, as this was close to the maximum distance measure from RCTs (0.027). This resulted in an exploration of 5% (*n* = 7) of possible matches. In Table 1, possible matches are shaded and final matches (lowest distance measures) are in bold. Overall, 2 matched sRWE studies were included in the analysis. Figures E1 and E2 in Appendix E show the digitized Kaplan-Meier curves for these matched sRWE studies. Appendix F shows that matching was reasonably robust to changes in covariate weights. A maximum distance measure of 0.055 was applied as a sensitivity analysis, as this was the maximum distance measure calculated between arms of cRWE. This resulted in an exploration of 23% (*n* = 30) of possible matches, which can be seen in Table G1 in Appendix G.

**Table 1:** Distance Metric between Single-Arm Observational Studies^a^

	Dong 2015^40^	Matsumoto 2007^41^	Catalano 2009^42^	Fuse 2008^43^	Suenaga 2008^44^	Fuse 2007^45^	Hochster 2003^46^	Yoshino 2007^47^
Bendell 2012 (1)^48^	**0.029**	0.273	0.048	0.134	0.077	0.126	0.043	0.057
Bendell 2012 (2)^48^	0.034	0.273	0.043	0.139	0.074	0.131	0.041	0.062
Hurwitz 2014^49^	0.151	0.156	0.158	0.211	0.171	0.248	0.169	0.179
Van Cutsem 2009 (1)^50^	0.051	0.234	0.092	0.093	0.040	0.085	0.073	0.018
Van Cutsem 2009 (2)^50^	0.034	0.232	0.085	0.082	0.044	0.074	0.083	0.018
Van Cutsem 2009 (3)^50^	0.047	0.221	0.096	0.072	0.045	0.064	0.094	**0.013**
Van Cutsem 2009 (4)^50^	0.051	0.224	0.095	0.079	0.045	0.071	0.087	0.017
Bennouna 2017 (1)^51^	0.058	0.227	0.069	0.110	0.073	0.102	0.089	0.041
Bennouna 2017 (2)^51^	0.208	0.115	0.208	0.175	0.204	0.212	0.242	0.191
Buchler 2014 (1)^52^	0.045	0.233	0.079	0.094	0.070	0.086	0.080	0.025
Buchler 2014 (2)^52^	0.052	0.213	0.088	0.082	0.050	0.072	0.091	0.014
Ocvirk 2011 (1)^53^	0.043	0.260	0.072	0.100	0.066	0.094	0.066	0.065
Ocvirk 2011 (2)^53^	0.051	0.226	0.097	0.080	0.047	0.062	0.096	0.044
Moriwaki 2012 (1)^54^	0.204	0.062	0.233	0.121	0.160	0.148	0.243	0.165
Moriwaki 2012 (2)^54^	0.184	0.099	0.228	0.132	0.129	0.169	0.211	0.150
Kotaka 2016^55^	0.068	0.200	0.095	0.072	0.033	0.064	0.104	0.034

a Columns refer to single-arm studies on the control arm, and rows refer to single-arm studies on the treatment arm. Shaded cells indicate a distance measure lower than the matching threshold. Bolded cells indicate the final matched studies.

**Figure 1:**
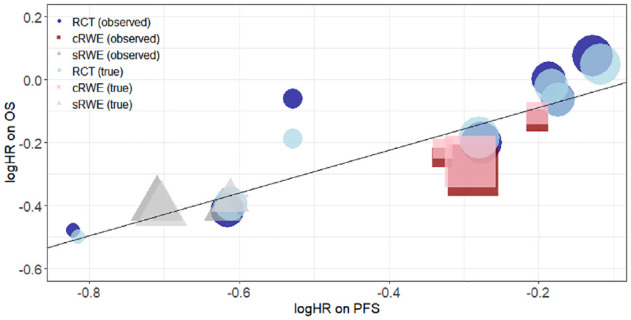
Scatterplot of logHRs on OS against logHRs on PFS. Dark blue circles, dark red squares, and dark gray triangles show observed treatment effects from RCTs, cRWE, and matched sRWE, respectively. Light blue circles, light red squares, and light gray triangles show true treatment effects estimated by the D&H model from RCTs, cRWE, and matched sRWE, respectively. The black line shows the linear relationship between logHR on PFS and logHR on OS obtained from the D&H model conducted using all sources of evidence. The size of points refers to the size of the study. cRWE, comparative real-world evidence; D&H, Daniels and Hughes; OS, overall survival; PFS, progression-free survival; RCT, randomized controlled trial; sRWE, single-arm real-world evidence.

Figure 1 presents a scatter plot of the observed and true treatment effects on PFS and OS, where the observed treatment effects ( $Y_{\mathrm{ji}}$ ) are those reported in the studies, and the true treatment effects ( $\delta_{\mathrm{ji}}$ ) are the underlying treatment effects estimated by the D&H model. The plot shows a possible strong positive relationship between treatment effects on PFS and OS, suggesting a potential valid surrogate relationship.

### D&H Model

**Figure 2:**
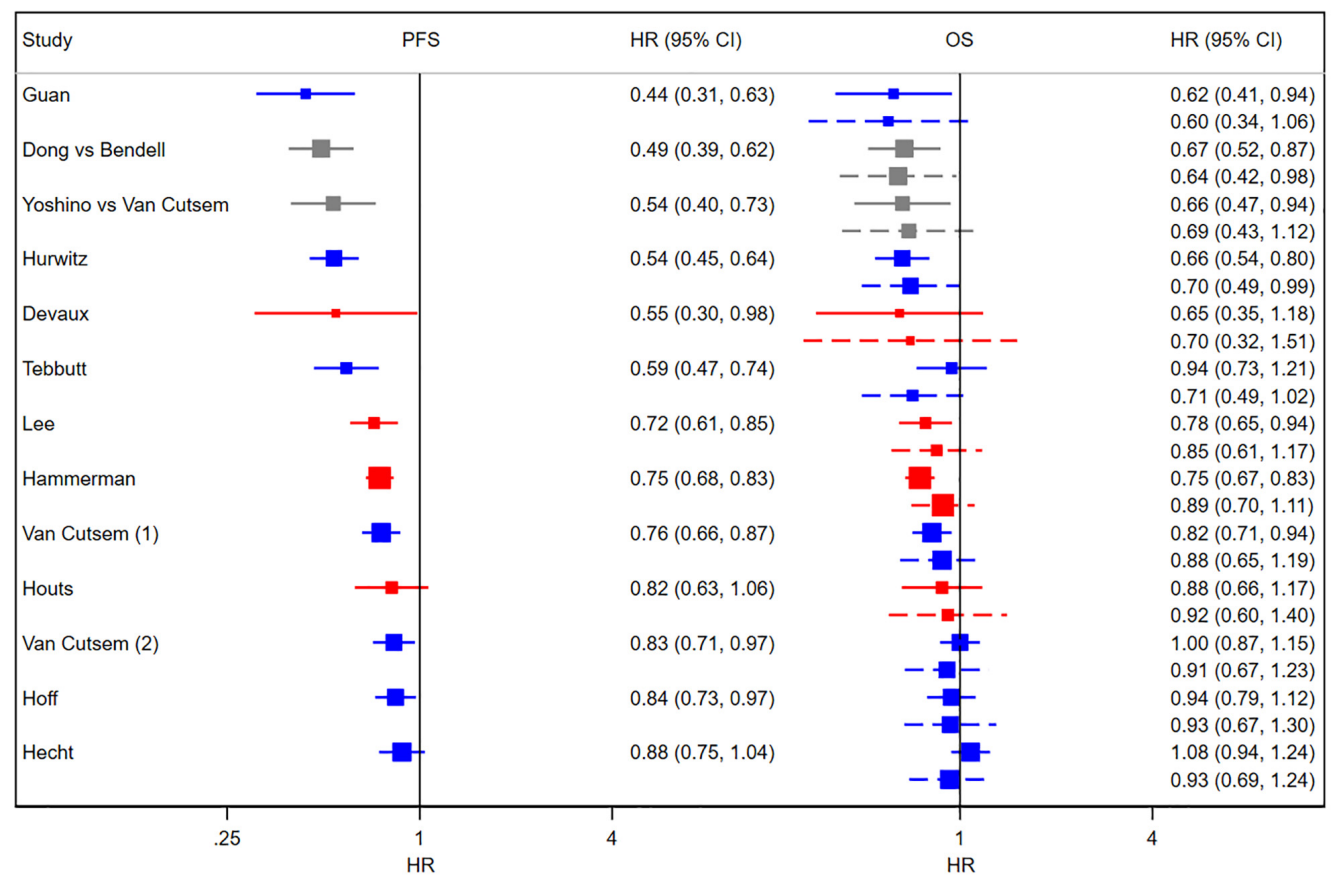
Forest plots of HRs from RCTs, cRWE, and sRWE. Left panel: PFS; right panel: OS. Solid lines show the observed 95% confidence intervals, and dashed lines show 95% predicted intervals obtained from cross-validation using D&H model. Blue shows RCTs, red shows cRWE, and gray shows matched sRWE. cRWE, comparative real-world evidence; D&H, Daniels and Hughes; HR, hazard ratio; OS, overall survival; PFS, progression-free survival; RCT, randomized controlled trial; sRWE, single-arm real-world evidence.

Table 2 shows results from the D&H model, for which the code is available in Appendix H and history plots suggesting convergence are available in Appendix I. While there are small changes in the point estimates for the surrogacy parameters with the addition of cRWE and matched sRWE, the overall conclusions on evidence for a surrogate relationship do not change with the addition of RWE. There is evidence of a surrogate relationship regardless of the type of evidence used, as the 95% CrIs for the intercept contain zero, the 95% CrIs for the slope do not contain zero, and the conditional variances are close to zero. Figure 2 illustrates this relationship, highlighting that studies with larger treatment effects on PFS generally also have larger treatment effects on OS.

**Table 2:** Surrogacy criteria obtained from D&H model applied to data from RCTs, cRWE, and matched sRWE^a^

	RCTs	RCTs and cRWE	RCTs, cRWE, and sRWE (Lower Matching)	RCTs, cRWE, and sRWE (Higher Matching)
$\lambda_{0}$	0.11 (−0.13, 0.34)	0.051 (−0.13, 0.23)	0.050 (−0.10, 0.20)	−0.056 (−0.25, 0.11)
$\lambda_{1}$	0.71 (0.12, 1.30)	0.69 (0.20, 1.17)	0.68 (0.31, 1.04)	0.47 (0.0012, 0.91)
$\psi_{2}^{2}$	0.0089 (0.0000, 0.11)	0.010 (0.0002, 0.051)	0.0088 (0.0003, 0.037)	0.013 (0.0004, 0.062)
Absolute discrepancy, median (range)	0.091 (0.0021, 0.27)	0.070 (0.0079, 0.30)	0.072 (0.0074, 0.28)	0.095 (0.015, 0.68)
$w_{{\hat{Y}}_{2j}}/w_{Y_{2j}}$ , median (range)	2.90 (1.76, 3.58)	2.09 (1.33, 2.40)	1.72 (1.28, 2.15)	1.77 (1.20, 2.78)

cRWE, comparative real-world evidence; D&H, Daniels and Hughes; OS, overall survival; PFS, progression-free survival; RCT, randomized controlled trial; sRWE, single-arm real-world evidence.

a The last 2 columns provide the results from the D&H model using a lower matching threshold (0.030) and higher matching threshold (0.055) for matching sRWE. The 2 rows provide the cross-validation results from the D&H model, where absolute discrepancy is the absolute difference between the observed logHR and the predicted logHR on OS, while $w_{{\hat{Y}}_{2j}}/w_{Y_{2j}}$ is the ratio of the width of the 95% predicted interval of the logHR on OS to the width of the 95% CI of the observed estimate of the logHR on OS.

The addition of cRWE to RCTs improved the precision of all 3 estimates for the surrogate relationship while having a minimal impact on the point estimates. Using RCT data alone resulted in the conditional variance 0.0089 (95% CrI: 0.0000, 0.11), while the addition of cRWE gave a conditional variance of 0.010 (95% CrI: 0.0002, 0.051). Thus, the addition of cRWE reduced uncertainty by 54% in terms of the CrI width. The addition of sRWE to RCTs and cRWE further improved precision of the intercept, slope, and conditional variance estimates. Overall, the addition of RWE improved the precision of the estimation of the intercept, slope, and conditional variance by 36%, 38%, and 67%, respectively, relative to using RCT data alone. Tables J1 and K1 in Appendices J and K, respectively, show that the results from the D&H model are robust to changes in prior distributions for the within-study correlation and the conditional standard deviation. Column 4 of Table 2 shows that using a higher maximum value for matching sRWE studies (based on the highest distance for cRWE of 0.055), and thus including more matched sRWE studies, did not result in further improvements in precision of the surrogacy parameters.

### BRMA (PNF)

Table 3 shows there was weaker evidence for a surrogate relationship using the BRMA PNF compared with the D&H model. Although the between-study correlation was relatively high (0.74; using all evidence), the CrI corresponding to the correlation was wide (95% CrI: 0.065, 0.97). Furthermore, the surrogacy criteria were not fully satisfied, as the 95% CrI for the slope contains zero when using RCTs alone and RCTs plus cRWE. This provides weaker evidence for an association between treatment effects on the surrogate endpoint and treatment effects on the final outcome. When including RCTs, cRWE, and matched sRWE, there was stronger evidence of an association, as shown by the positive value for the slope with a 95% CrI that excluded zero and the conditional variance, which was reduced together with the upper end of the 95% CrI. However, the estimate for the slope was obtained with greater uncertainty compared with the slope estimated from the D&H model when using RCTs, cRWE, and matched sRWE. Such differences in results between the models could be a result of the assumption of random effects in the BRMA PNF model. When assuming that normal random effects is appropriate, greater borrowing of information can lead to more precise estimates. However, when this assumption is violated, the model can lead to overshrinkage of the true effects, thus potentially reducing the between-studies correlation.^56,57^ This can be observed in Figure L1 in Appendix L, where the true effects (obtained from BRMA PNF) in the bubble plot are more shrunken toward the mean, in particular for PFS, compared with those obtained from the D&H model (depicted in Figure 1). This can lead to bias and reduce precision of estimates for surrogacy parameters.

**Table 3:** Surrogacy Criteria Obtained from the BRMA PNF Model Applied to Data from RCTs, cRWE, and Matched sRWE^a^

	RCTs	RCTs, cRWE	RCTs, cRWE, sRWE	RCTs, cRWE, sRWE (Accounting for Bias)
$d_{1}$	−0.36 (−0.61, −0.13)	−0.34 (−0.49, −0.21)	−0.39 (−0.53, −0.26)	−0.36 (−0.55, −0.18)
$d_{2}$	−0.14 (−0.35, 0.046)	−0.18 (−0.31, −0.052)	−0.21 (−0.33, −0.093)	−0.14 (−0.30, 0.0075)
ρ	0.75 (−0.25, 0.99)	0.66 (−0.30, 0.97)	0.74 (0.065, 0.97)	0.73 (−0.22, 0.98)
$\tau_{1}$	0.25 (0.11, 0.60)	0.18 (0.079, 0.36)	0.20 (0.11, 0.36)	0.19 (0.091, 0.39)
$\tau_{2}$	0.19 (0.073, 0.48)	0.15 (0.072, 0.31)	0.16 (0.080, 0.30)	0.15 (0.054, 0.32)
$\lambda_{0}$	0.054 (−0.22, 0.33)	0.0068 (−0.26, 0.28)	0.012 (−0.19, 0.21)	0.048 (−0.19, 0.29)
$\lambda_{1}$	0.54 (−0.16, 1.31)	0.54 (−0.25, 1.40)	0.56 (0.041, 1.12)	0.52 (−0.14, 1.23)
$\psi_{2}^{2}$	0.013 (0.0008, 0.099)	0.011 (0.0014, 0.048)	0.0099 (0.008, 0.037)	0.0084 (0.0005, 0.051)
$R^{2}$	0.57 (0.0043, 0.98)	0.43 (0.0023, 0.94)	0.55 (0.0014, 0.94)	0.54 (0.0046, 0.97)
$\alpha_{1}$	—	—	—	0.036 (−0.40, 0.47)
$\alpha_{2}$	—	—	—	−0.12 (−0.49, 0.27)
$\beta_{1}$	—	—	—	−0.31 (−1.68, 1.07)
$\beta_{2}$	—	—	—	−0.27 (−1.60, 1.04)
Absolute discrepancy, median (range)	0.16 (0.017, 0.26)	0.16 (0.0046, 0.23)	0.13 (0.0036, 0.24)	0.16 (0.011, 0.28)
$w_{{\hat{Y}}_{2j}}/w_{Y_{2j}}$ , median (Range)	2.74 (1.61, 3.30)	2.09 (1.15, 2.49)	1.72 (1.14, 2.28)	1.80 (1.17, 3.50)

BRMA, bivariate random-effects meta-analysis; CI, confidence interval; cRWE, comparative real-world evidence; D&H, Daniels and Hughes; OS, overall survival; PNF, product normal formulation; RCT, randomized controlled trial; sRWE, single-arm real-world evidence.

a The last 2 rows provide the cross-validation results from the BRMA PNF model, where the absolute discrepancy is the absolute difference between the observed logHR and the predicted logHR on OS, while $w_{{\hat{Y}}_{2j}}/w_{Y_{2j}}$ is the ratio of the width of the 95% predicted interval of the logHR on OS to the width of the 95% CI of the observed estimate of the logHR on OS.

Despite the slightly weaker evidence supporting a surrogate relationship compared with the D&H model, the addition of RWE generally improved the precision of estimates obtained from the BRMA PNF model. Using RCT data alone, the correlation was 0.75 (95% CrI: −0.25, 0.99), whereas when using all sources of evidence, the correlation was 0.74 (95% CrI: 0.065, 0.97). Thus, the addition of RWE reduced uncertainty by 27%, while there was little change in the point estimate. Tables J2 and K2 highlight that the BRMA PNF model is also robust to the assumptions about the within-study correlation and the choice of prior distributions for the between-studies heterogeneity parameters.

### Accounting for Bias

Table 3 shows results from the BRMA PNF model accounting for bias, described in the “Accounting for Bias” section. Relative to the model including RWE without accounting for bias, there was no improvement in the precision of the estimation of the intercept, slope, or conditional variance. In addition, the 95% CrI for the slope contained zero when using the model accounting for bias, providing weaker evidence for the association between treatment effects on the surrogate endpoint and treatment effects on the final outcome. However, relative to the model including RCT data only, when including RWE and accounting for bias, there was improvement in the precision of estimation of all surrogacy parameters while point estimates remained similar. For example, when using RCT data alone, the between-studies correlation was estimated to be 0.75 (95% CrI: −0.25, 0.99), but when RWE was included accounting for bias, the between-studies correlation was estimated at 0.73 (95% CrI: −0.22, 0.98). This indicated that the addition of RWE while accounting for bias resulted in a 3% improvement in precision of the between-studies correlation, while there was little change in the point estimate.

### Cross-validation

The last 2 rows of Table 2 show the results of cross-validation using the D&H model. The median absolute discrepancy between the predicted and observed treatment effects on OS decreased with the addition of cRWE and slightly increased with the further addition of sRWE. Table 2 also shows that the width of the predicted interval, relative to the observed CI, fell with the addition of cRWE (2.90 to 2.09) and sRWE (2.09 to 1.72), indicating that the precision of prediction improved with the addition of cRWE and matched sRWE. Cross-validation for the BRMA PNF model showed similar results (Table 3). Column 4 of Table 2 shows that relaxing the threshold for matching sRWE studies by using a higher maximum distance value, and thus including more matched sRWE studies in the analysis, resulted in slightly poorer accuracy and precision of predictions when using the D&H model.

## Discussion

When existing clinical trial data are limited, surrogate endpoint validation may fail. As a result, new therapies may not receive conditional marketing authorization or, if approved, may still fail at the health technology assessment (HTA) decision-making stage, by HTA agencies such as the National Institute for Health and Care Excellence (NICE).^2,3^ In this article, we provide an approach for using RWE to strengthen the evidence base for surrogate endpoint validation.

When including RWE alongside RCT data, it is important to carefully consider the quality of studies to avoid excess bias. When selecting RWE, we aimed to minimize bias by using a strict matching threshold for sRWE and by including only cRWE, which adjusted for covariates. In the motivating example, the inclusion of RWE improved the precision of estimation of the surrogacy parameters without substantially changing their point estimates. This implies that in this example, inclusion of RWE reduced the uncertainty around the surrogacy parameter estimates without inducing bias on the surrogacy parameter estimates. However, inclusion of RWE could result in substantially different point estimates for the surrogacy parameters, which could imply that the addition of RWE induced bias on the surrogacy parameter estimates. In this case, any improvements in precision should be interpreted with caution, and the quality of the RWE included should be assessed to determine whether excess bias has been appropriately accounted for.

Despite our careful consideration given to the selection of RWE, there are several limitations of this research. Inclusion of sRWE relied on digitizing Kaplan-Meier curves. However, such curves are not always published, and thus, potentially useful studies would not be included. One method to overcome this issue is to extract median survival times for the surrogate and final outcomes and use an exponential hazard assumption, as proposed by Schmitz et al.,^22^ to obtain treatment effects. However, our preliminary analysis showed that the assumption of exponential hazard did not provide a good approximation in this case study.

Matching of sRWE was based on study-level covariates and thus was prone to bias, as patients were not randomized or matched at the individual level, and therefore, the exchangeability assumption may have been violated.^58^ This bias was exacerbated by matching on only 5 covariates, when the consensus statement recommended 10 additional characteristics. However, these variables were not reported in the included studies. While acquisition of IPD would allow for use of complex methods such as propensity scoring to better adjust for measured covariates, IPD is often unavailable. For example, in HTA submissions, the manufacturer will have access to IPD from trials of their own technology; however, they are likely to have access to only aggregate data from studies of competitor technologies. Thus, in the absence of IPD, we propose matching sRWE based on aggregate-level data to permit inclusion of potentially useful single-arm studies.

The matched sRWE studies were analyzed according to the Cox proportional hazards model. It is possible that the assumption of proportional hazards was violated for some matched sRWE studies. However, all RCTs and cRWE studies in the analysis were analyzed using the log-rank test or Cox proportional hazards model, both of which assume proportional hazards. Visual inspection of Kaplan-Meier curves from RCTs and cRWE showed that some studies appeared to exhibit nonproportional hazards. Therefore, if accounting for nonproportional hazards present in sRWE studies, nonproportional hazards present in RCTs and cRWE studies should also be considered. This is a limitation often present in the meta-analysis of time-to-event outcomes, even when only RCT data are included. However, this issue is beyond the scope of this article and will be investigated in future work.

While RWE can increase the evidence base, it has limitations compared with RCTs. For example, despite using adjusted HRs and matching for cRWE and sRWE, RWE studies are likely to suffer from residual confounding. Furthermore, differences in data collection and evaluation of endpoints could make the treatment effects obtained from RCTs and RWE unsuitable for synthesis in a single meta-analysis.^59^ For example, participants of RCTs are followed up for a predefined period of time, and progression is assessed using predefined quantitative measures.^60^ However, in RWE, information is recorded as patients attend appointments and progression is based on clinical interpretation of imaging. Furthermore, RCTs frequently define time zero as time from randomization, whereas RWE studies define time zero as time from initiation of treatment.^61^ However, a study in oncology found that after adjusting for potential confounders, endpoints such as real-world PFS from RWE were similar to those observed in RCTs of participants given the same treatment.^61^

While the BRMA PNF model was extended to account for potential systematic differences in treatment effects between data sources, all sources of evidence contributed the same weight to the model, suggesting that RCTs, cRWE, and matched sRWE were of equivalent quality. However, RCTs have traditionally been considered the gold standard of evidence, as they provide more reliable sources of information about treatment effects compared with RWE.^62^ Further methodological research is carried out to allow for accounting for such differences in quality.

Finally, while the methods detailed in this article can be used to investigate different surrogate endpoints in different disease areas, the improvements in precision observed in the motivating example of PFS as a surrogate endpoint for OS in mCRC cannot be guaranteed in other disease areas or surrogate endpoints. The degree of improvement in precision will depend on a number of factors, including the quantity and heterogeneity of available evidence and the true underlying surrogacy pattern.

## Conclusions

RWE can be used to improve the precision of estimates for surrogate endpoint validation relative to using RCT data alone. The addition of RWE to RCT data also allows for more precise predictions to be made of the treatment effects on the final clinical outcome based on the treatment effect measured on the surrogate endpoint. When incorporated in a decision-modeling framework, such improved estimates can lead to cost-effectiveness estimates being obtained with reduced uncertainty, which can in turn lead to more robust policy decisions.

## Supplemental Material

sj-bib-2-mdm-10.1177_0272989X231162852 – Supplemental material for Using Bayesian Evidence Synthesis Methods to Incorporate Real-World Evidence in Surrogate Endpoint Evaluation Supplemental material, sj-bib-2-mdm-10.1177_0272989X231162852 for Using Bayesian Evidence Synthesis Methods to Incorporate Real-World Evidence in Surrogate Endpoint Evaluation by Lorna Wheaton, Anastasios Papanikos, Anne Thomas and Sylwia Bujkiewicz in Medical Decision Making

sj-docx-1-mdm-10.1177_0272989X231162852 – Supplemental material for Using Bayesian Evidence Synthesis Methods to Incorporate Real-World Evidence in Surrogate Endpoint Evaluation Supplemental material, sj-docx-1-mdm-10.1177_0272989X231162852 for Using Bayesian Evidence Synthesis Methods to Incorporate Real-World Evidence in Surrogate Endpoint Evaluation by Lorna Wheaton, Anastasios Papanikos, Anne Thomas and Sylwia Bujkiewicz in Medical Decision Making

sj-tex-3-mdm-10.1177_0272989X231162852 – Supplemental material for Using Bayesian Evidence Synthesis Methods to Incorporate Real-World Evidence in Surrogate Endpoint Evaluation Supplemental material, sj-tex-3-mdm-10.1177_0272989X231162852 for Using Bayesian Evidence Synthesis Methods to Incorporate Real-World Evidence in Surrogate Endpoint Evaluation by Lorna Wheaton, Anastasios Papanikos, Anne Thomas and Sylwia Bujkiewicz in Medical Decision Making

sj-tex-4-mdm-10.1177_0272989X231162852 – Supplemental material for Using Bayesian Evidence Synthesis Methods to Incorporate Real-World Evidence in Surrogate Endpoint Evaluation Supplemental material, sj-tex-4-mdm-10.1177_0272989X231162852 for Using Bayesian Evidence Synthesis Methods to Incorporate Real-World Evidence in Surrogate Endpoint Evaluation by Lorna Wheaton, Anastasios Papanikos, Anne Thomas and Sylwia Bujkiewicz in Medical Decision Making
