# Supplementary material for: Using Bayesian Evidence Synthesis Methods to Incorporate Real-World Evidence in Surrogate Endpoint Evaluation
Source: Med Decis Making. 2023 Mar 30;43(5):539–52. doi: 10.1177/0272989X231162852 (PMC10336701; doi:10.1177/0272989X231162852)
Supplement: sj-docx-1-mdm-10.1177_0272989X231162852 – Supplemental material for Using Bayesian Evidence Synthesis Methods to Incorporate Real-World Evidence in Surrogate Endpoint Evaluation [file sj-docx-1-mdm-10.1177_0272989X231162852.docx]

**Additional File 1**

**List of Contents**

**Appendix A: Complete Dataset**

**Appendix B: Covariate Adjustment in cRWE studies**

**Appendix C: Baseline Characteristics from Consensus Statement**

**Appendix D: Example of matching sRWE studies**

**Appendix E: Digitized Kaplan-Meier curves for matched sRWE studies**

**Appendix F: Sensitivity analysis to covariate weights when matching sRWE studies**

**Appendix G: Sensitivity analysis to maximum distance measure when matching sRWE studies**

**Appendix H: R code for data analysis**

**Appendix I: History plots**

**Appendix J: Results of D&H and BRMA PNF models using alternative within-study correlation**

**Appendix K: Results of D&H and BRMA PNF models using Unif(0,100) for SD**

**Appendix L: Bubble Plot for BRMA PNF**

**Appendix A: Complete Dataset**

Table A1: Studies included in final analysis

| Study | Study Type | PFS | SE PFS | OS | SE OS |
| --- | --- | --- | --- | --- | --- |
| [Guan 2011](https://www.ncbi.nlm.nih.gov/pmc/articles/PMC4012268/) | RCT | -0.821 | 0.181 | -0.478 | 0.214 |
| [Hurwitz 2004](https://pubmed.ncbi.nlm.nih.gov/15175435/) | RCT | -0.616 | 0.09 | -0.416 | 0.1 |
| [Hecht 2011](http://nlp.case.edu/public/data/TargetedToxicity_JCOFullText/SVM_text_classifier_training/training/positive/1_419.html) | RCT | -0.128 | 0.084 | 0.077 | 0.071 |
| [Hoff 2012](https://pubmed.ncbi.nlm.nih.gov/22965965/) | RCT | -0.174 | 0.075 | -0.062 | 0.089 |
| [Tebbutt 2010](https://pubmed.ncbi.nlm.nih.gov/20516443/) | RCT | -0.528 | 0.119 | -0.06 | 0.129 |
| [Van Cutsem 2011](https://pubmed.ncbi.nlm.nih.gov/21464401/) | RCT | -0.186 | 0.077 | 0 | 0.073 |
| [Van Cutsem 2012](https://pubmed.ncbi.nlm.nih.gov/22949147/) | RCT | -0.277 | 0.07 | -0.202 | 0.072 |
| [Hammerman 2014](https://www.tandfonline.com/doi/full/10.3109/0284186X.2014.958532) | cRWE | -0.288 | 0.0509 | -0.288 | 0.0539 |
| [Lee 2017](https://pubmed.ncbi.nlm.nih.gov/27885818/) | cRWE | -0.329 | 0.0875 | -0.248 | 0.0969 |
| [Devaux 2019](https://www.ncbi.nlm.nih.gov/pmc/articles/PMC6390115/) | cRWE | -0.603 | 0.299 | -0.435 | 0.307 |
| [Houts 2019](https://link.springer.com/article/10.1007/s12029-017-0017-8) | cRWE | -0.202 | 0.135 | -0.129 | 0.148 |
| [Dong 2015](https://link.springer.com/article/10.1007/s13277-015-3492-1) vs [Bendell 2012](https://academic.oup.com/oncolo/article/17/12/1486/6403280?login=true) | sRWE | -0.71 | 0.119 | -0.4 | 0.133 |
| [Yoshino 2007](https://academic.oup.com/jjco/article/37/9/686/825295?login=true) vs [Van Cutsem 2009](https://pubmed.ncbi.nlm.nih.gov/19406901/) | sRWE | -0.622 | 0.156 | -0.414 | 0.177 |

**Appendix B: Covariate Adjustment in cRWE studies**

Table B1: Adjustment for confounders in cRWE studies

| Potential Confounder | Hammerman 2014 | | Lee 2017 | | Devaux 2019 | | Houts 2019 | |
| --- | --- | --- | --- | --- | --- | --- | --- | --- |
|  | PFS | OS | PFS | OS | PFS | OS | PFS | OS |
| Age | ✓ | ✓ | 🗶 | ✓ | ✓ | ✓ | ✓ | ✓ |
| BMI | 🗶 | 🗶 | 🗶 | 🗶 | 🗶 | 🗶 | ✓ | ✓ |
| Charlson's comorbidity score | ✓ | ✓ | 🗶 | 🗶 | 🗶 | 🗶 | 🗶 | 🗶 |
| Exposure to Irinotecan and Oxaliplatin | ✓ | ✓ | 🗶 | 🗶 | ✓ | ✓ | ✓ | ✓ |
| Line of treatment | 🗶 | 🗶 | 🗶 | 🗶 | ✓ | ✓ | 🗶 | 🗶 |
| Liver metastasis? | 🗶 | 🗶 | 🗶 | 🗶 | 🗶 | 🗶 | ✓ | ✓ |
| Lung metastasis? | 🗶 | 🗶 | 🗶 | 🗶 | 🗶 | 🗶 | ✓ | ✓ |
| Number of metastases | 🗶 | 🗶 | ✓ | ✓ | ✓ | ✓ | 🗶 | 🗶 |
| Performance status (e.g. ECOG/WHO) | 🗶 | 🗶 | ✓ | ✓ | ✓ | ✓ | ✓ | ✓ |
| Primary resection | 🗶 | 🗶 | ✓ | ✓ | ✓ | ✓ | 🗶 | 🗶 |
| Race | 🗶 | 🗶 | 🗶 | 🗶 | 🗶 | 🗶 | ✓ | ✓ |
| RAS/BRAF Mutation status | 🗶 | 🗶 | 🗶 | 🗶 | ✓ | ✓ | 🗶 | 🗶 |
| Sex | ✓ | ✓ | 🗶 | 🗶 | ✓ | ✓ | ✓ | ✓ |
| Stage at diagnosis | 🗶 | 🗶 | 🗶 | 🗶 | 🗶 | 🗶 | ✓ | ✓ |
| Surgery of metastases? | 🗶 | 🗶 | 🗶 | 🗶 | ✓ | ✓ | 🗶 | 🗶 |
| Synchronous? | 🗶 | 🗶 | 🗶 | 🗶 | ✓ | ✓ | 🗶 | 🗶 |
| Tumour location | 🗶 | 🗶 | 🗶 | 🗶 | ✓ | ✓ | 🗶 | 🗶 |

**Appendix C: Baseline Characteristics from Consensus Statement**

Table C1: Baseline characteristics from consensus statement

| Characteristic | Recommendation |
| --- | --- |
| Age | Recommended |
| Performance status | Recommended |
| Location primary tumour | Recommended |
| Surgery primary tumour | Recommended |
| Prior chemotherapy | Recommended |
| Number of metastatic sites | Recommended |
| Liver-only disease | Recommended |
| Liver involvement | Recommended |
| Surgery metastases | Recommended |
| Synchronous vs metachronous metastases | Recommended |
| (K)RAS mutation status | Recommended |
| BRAF mutation status | Recommended |
| MSI/MMR status | Recommended |
| Number of prior treatment lines | Recommended (later line trials) |
| Gender | Suggested |
| Race/ethnicity | Suggested |
| Prior radiotherapy | Suggested |
| Stage at first diagnosis | Suggested |
| Tumour differentiation | Suggested |
| Lactate dehydrogenase (LDH) | Suggested |
| Alkaline phosphatase (ALP) | Suggested |
| Carcinoembryonic antigen (CEA) | Suggested |
| Albumin | Suggested |
| Platelet count | Suggested |
| Initially resectable metastatic disease | Suggested |
| Lung-only disease | Suggested |
| Peritoneal disease | Suggested |
| Number of metastases | Suggested |
| Comorbidity or fit vs unfit patient | Suggested |
| Weight/BMI | Suggested |
| Weight loss | Suggested (later line trials) |
| Symptomatic disease | Suggested (later line trials) |
| Truly refractory versus “just discontinued” prior treatments | Suggested (later line trials) |
| Time from diagnosis mCRC to start of treatment | Suggested (later line trials) |
| Response and PFS on prior treatments | Suggested (later line trials) |
| Time from last treatment to start of trial | Suggested (later line trials) |

**Appendix D: Example of matching sRWE studies**

Data example of matching single arm studies:

Table D1: Example of baseline characteristics obtained from sRWE studies

| Study | Treatment | Treatment Line (mean) | Age (median) | ECOG/WHO Score (mean) | Tumour Location (proportion in Rectum) | Female (%) |
| --- | --- | --- | --- | --- | --- | --- |
| Bendell 2012 (1) | FOLFOX + Bevacizumab | 1 | 61 | 0.65 | 0.74 | 43 |
| Dong 2015 | FOLFIRI | 1 | 57 | 0.55 | 0.71 | 39 |

Formula for scaling baseline characteristics between 0 and 1:

$$x_{normalised}=\frac{x-x_{min}}{x_{max}-x_{min}}$$

Example of scaling baseline characteristics between 0 and 1:

Treatment line:

$$\frac{1-1}{3-1}=0$$

Age:

$$\frac{61-18}{100-18}=0.52$$

ECOG/WHO score:

$$\frac{0.65-0}{3-0}=0.2167$$

Tumour location 0.74 is already expressed as a proportion between 0 and 1.

Sex 0.43 is already expressed as a proportion between 0 and 1.

Table D2: Example of matching sRWE studies

| Study | Treatment | Treatment Line | Age | ECOG/WHO Score | Tumour Location | Female |
| --- | --- | --- | --- | --- | --- | --- |
| Assumed range | - | 1-3 | 18-100 | 0-3 | Colon (0) vs Rectum (1) | 0-100 |
| Weight | - | 2 | 2 | 2 | 2 | 1 |
| Bendell 2012 (1) | FOLFOX + Bevacizumab | 0 | 0.52 | 0.22 | 0.74 | 0.43 |
| Dong 2015 | FOLFIRI | 0 | 0.48 | 0.18 | 0.71 | 0.39 |
| $\Delta$ Individual characteristics | - | 0 | 0.04 | 0.04 | 0.03 | 0.04 |

Calculating weighted average between two studies to obtain overall distance measure:

$$\Delta_{tot}=\frac{\left( 2\times0 \right)+\left( 2\times0.04 \right)+\left( 2\times0.04 \right)+\left( 2\times0.03 \right)+(1\times0.04)}{9}=0.0289$$

**Appendix E: Digitized Kaplan-Meier Curves for matched single-arm RWE**

**
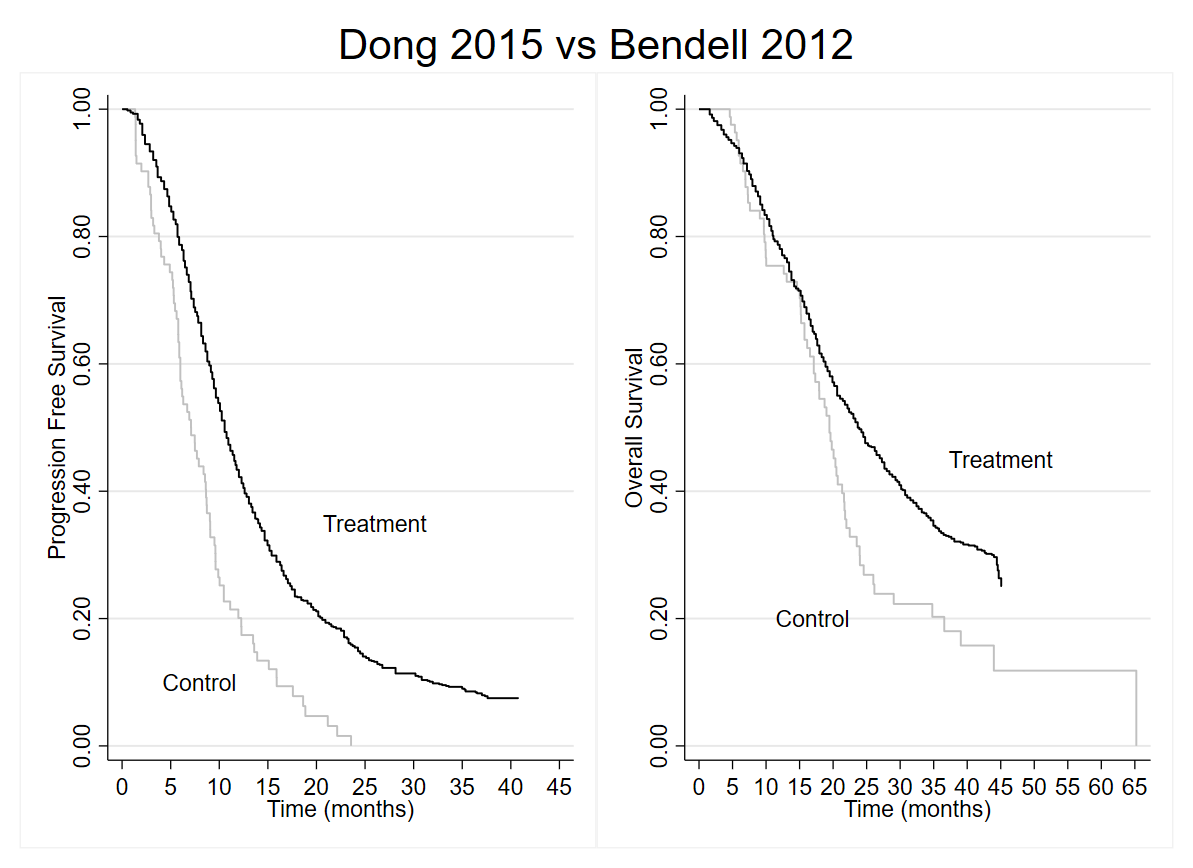
**

Figure E1: Digitized Kaplan-Meier curves for PFS and OS for Dong 2015 vs Bendell 2012 matched sRWE study


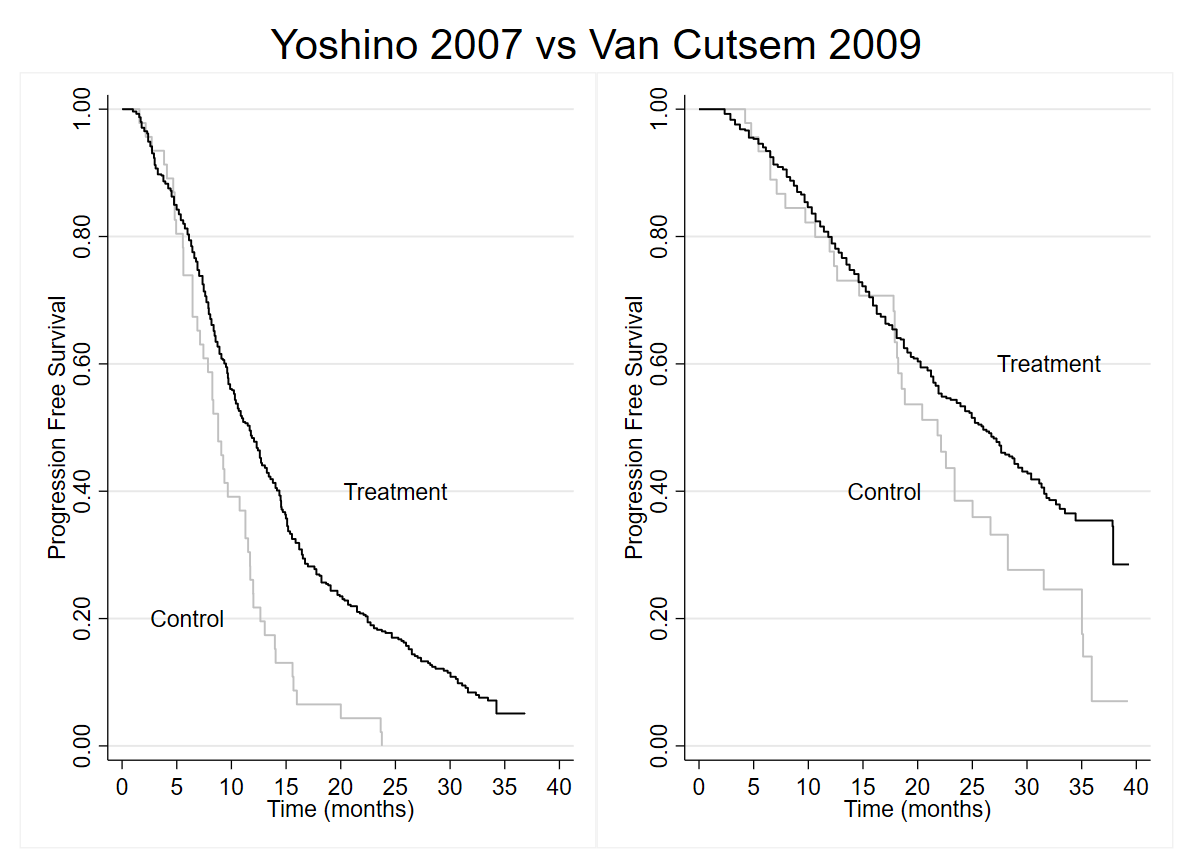


Figure E2: Digitized Kaplan-Meier curves for PFS and OS for Yoshino 2007 vs Van Cutsem 2009 matched sRWE study

**Appendix F: Sensitivity Analysis to covariate weights when matching sRWE studies**

Five covariates were reported in all sRWE studies. Sex was rated “very important” by 50% of experts, treatment line was rated “very important” by 80% of experts and tumour location, age and performance score were rated “very important” by 90% of experts. Tumour location was rated “not important” by 3% of experts whereas age and performance score were rated “not important” by zero experts. Therefore, with equal ranking, age and performance score are assigned a weight of 4.5, tumour location weight 3, treatment line weight 2 and sex weight 1. The results of matching according to these weights can be seen in Table C1.

Table F1: Distance Metric between Single Arm Observational Studies using alternative weightings: Columns refer to single-arm studies on the control arm and rows refer to single-arm studies on the treatment arm. Shaded cells indicate a distance measure lower than the matching threshold. Bolded cells indicate the final matched studies.

|  | Dong 2015 | Matsumoto 2007 | Catalano 2009 | Fuse 2008 | Suenaga 2008 | Fuse 2007 | Hochster 2003 | Yoshino 2007 |
| --- | --- | --- | --- | --- | --- | --- | --- | --- |
| Bendell 2012 (1) | 0.033 | 0.221 | 0.052 | 0.133 | 0.080 | 0.132 | 0.038 | 0.059 |
| Bendell 2012 (2) | 0.038 | 0.218 | 0.045 | 0.137 | 0.076 | 0.136 | 0.037 | 0.063 |
| Hurwitz 2014 (3) | 0.115 | 0.147 | 0.116 | 0.188 | 0.141 | 0.213 | 0.124 | 0.141 |
| Van Cutsem 2009 (1) | 0.052 | 0.177 | 0.094 | 0.086 | 0.042 | 0.084 | 0.070 | **0.015** |
| Van Cutsem 2009 (2) | 0.039 | 0.178 | 0.094 | 0.075 | 0.042 | 0.074 | 0.080 | 0.018 |
| Van Cutsem 2009 (3) | 0.052 | 0.166 | 0.105 | 0.064 | 0.042 | 0.062 | 0.091 | **0.015** |
| Van Cutsem 2009 (4) | 0.055 | 0.168 | 0.101 | 0.072 | 0.043 | 0.071 | 0.083 | 0.017 |
| Bennouna 2017 (1) | 0.065 | 0.175 | 0.066 | 0.116 | 0.079 | 0.115 | 0.084 | 0.050 |
| Bennouna 2017 (2) | 0.176 | 0.117 | 0.163 | 0.165 | 0.178 | 0.190 | 0.197 | 0.161 |
| Buchler 2014 (1) | 0.047 | 0.186 | 0.079 | 0.098 | 0.072 | 0.096 | 0.069 | 0.031 |
| Buchler 2014 (2) | 0.057 | 0.160 | 0.089 | 0.081 | 0.044 | 0.078 | 0.083 | 0.017 |
| Ocvirk 2011 (1) | 0.049 | 0.200 | 0.089 | 0.083 | 0.061 | 0.085 | 0.072 | 0.066 |
| Ocvirk 2011 (2) | 0.064 | 0.169 | 0.111 | 0.072 | 0.040 | 0.056 | 0.097 | 0.052 |
| Moriwaki 2012 (1) | 0.164 | 0.053 | 0.189 | 0.092 | 0.112 | 0.112 | 0.190 | 0.124 |
| Moriwaki 2012 (2) | 0.144 | 0.084 | 0.190 | 0.095 | 0.090 | 0.120 | 0.167 | 0.106 |
| Kotaka 2016 | 0.080 | 0.139 | 0.104 | 0.065 | **0.025** | 0.064 | 0.104 | 0.042 |

**Appendix G: Sensitivity analysis to maximum distance measure when matching sRWE studies**

Table G1: Distance metric between single-arm observational studies using 0.055 as the maximum distance measure for matching single-arm studies: Columns refer to single-arm studies on the control arm and rows refer to single-arm studies on the treatment arm. Shaded cells indicate a distance measure lower than the matching threshold. Bolded cells indicate the final matched studies.

|  | Dong 2015 | Matsumoto 2007 | Catalano 2009 | Fuse 2008 | Suenaga 2008 | Fuse 2007 | Hochster 2003 | Yoshino 2007 |
| --- | --- | --- | --- | --- | --- | --- | --- | --- |
| Bendell 2012 (1) | **0.029** | 0.273 | 0.048 | 0.134 | 0.077 | 0.126 | 0.043 | 0.057 |
| Bendell 2012 (2) | 0.034 | 0.273 | 0.043 | 0.139 | 0.074 | 0.131 | **0.041** | 0.062 |
| Hurwitz 2014 | 0.151 | 0.156 | 0.158 | 0.211 | 0.171 | 0.248 | 0.169 | 0.179 |
| Van Cutsem 2009 (1) | 0.051 | 0.234 | 0.092 | 0.093 | 0.040 | 0.085 | 0.073 | 0.018 |
| Van Cutsem 2009 (2) | 0.034 | 0.232 | 0.085 | 0.082 | 0.044 | 0.074 | 0.083 | 0.018 |
| Van Cutsem 2009 (3) | 0.047 | 0.221 | 0.096 | 0.072 | 0.045 | 0.064 | 0.094 | **0.013** |
| Van Cutsem 2009 (4) | 0.051 | 0.224 | 0.095 | 0.079 | 0.045 | 0.071 | 0.087 | 0.017 |
| Bennouna 2017 (1) | 0.058 | 0.227 | 0.069 | 0.110 | 0.073 | 0.102 | 0.089 | 0.041 |
| Bennouna 2017 (2) | 0.208 | 0.115 | 0.208 | 0.175 | 0.204 | 0.212 | 0.242 | 0.191 |
| Buchler 2014 (1) | 0.045 | 0.233 | 0.079 | 0.094 | 0.070 | 0.086 | 0.080 | 0.025 |
| Buchler 2014 (2) | 0.052 | 0.213 | 0.088 | 0.082 | 0.050 | 0.072 | 0.091 | 0.014 |
| Ocvirk 2011 (1) | 0.043 | 0.260 | 0.072 | 0.100 | 0.066 | 0.094 | 0.066 | 0.065 |
| Ocvirk 2011 (2) | 0.051 | 0.226 | 0.097 | 0.080 | 0.047 | 0.062 | 0.096 | 0.044 |
| Moriwaki 2012 (1) | 0.204 | 0.062 | 0.233 | 0.121 | 0.160 | 0.148 | 0.243 | 0.165 |
| Moriwaki 2012 (2) | 0.184 | 0.099 | 0.228 | 0.132 | 0.129 | 0.169 | 0.211 | 0.150 |
| Kotaka 2016 | 0.068 | 0.200 | 0.095 | 0.072 | **0.033** | 0.064 | 0.104 | 0.034 |

**Appendix H: R Code for data analysis**

######################################################

# D&H Model for RCTs, cRWE and sRWE

######################################################

# Clear Environment

rm(list = ls())

# Set working directory

work.dir <- "…"

setwd(work.dir)

# Load and check R2WinBUGS package

library(R2WinBUGS)

packageVersion("R2WinBUGS")

# Go to WinBUGS directory

bd <- "…"

# Create data for WinBUGS

data <- list(num=13, Y=structure(.Data=c(-0.821, -0.616, -0.128, -0.174,

-0.528, -0.186, -0.277, -0.288, -0.329, -0.603, -0.202, -0.71,

-0.622, -0.478, -0.416, 0.077, -0.062, -0.06, 0, -0.202,

-0.288, -0.248, -0.435, -0.129, -0.4, -0.414),

.Dim=c(13,2)), se=structure(.Data=c(0.181, 0.09, 0.084, 0.075,

0.119, 0.077, 0.07, 0.0509, 0.0875, 0.299, 0.135, 0.119,

0.156, 0.214, 0.1, 0.071, 0.089, 0.129, 0.073, 0.072, 0.0539,

0.0969, 0.307, 0.148, 0.133, 0.177), .Dim=c(13,2)))

bugs.data(data)

# Create text file for bugs code for D&H model

code<- "model{

rho_w ~ dunif(0, 0.999)

for (i in 1:num){

Prec_w[i,1:2, 1:2] <- inverse(sigma[i, 1:2, 1:2])

sigma[i,1,1] <- pow(se[i,1],2)

sigma[i,2,2] <- pow(se[i,2],2)

sigma[i,1,2] <- sqrt(sigma[i,1,1])*sqrt(sigma[i,2,2])*rho_w

sigma[i,2,1] <- sqrt(sigma[i,1,1])*sqrt(sigma[i,2,2])*rho_w

}

#Bivariate model for surrogacy

for (i in 1:num) {

Y[i, 1:2] ~ dmnorm(delta[i, 1:2], Prec_w[i, 1:2, 1:2])

# Daniels and Hughes formulation for between-studies model

delta[i,1] ~ dnorm(0.0, 0.001)

delta[i,2] ~ dnorm(eta2[i], prec2)

eta2[i] <- lambda20 + lambda21*delta[i,1]

}

# Prior distributions

lambda20 ~ dnorm(0.0, 1.0E-3)

lambda21 ~ dnorm(0.0, 1.0E-3)

psi.2 ~ dunif(0,2)

psi2.sq <- pow(psi.2,2)

prec2 <- 1/psi2.sq

}"

writeLines(code,"code.txt")

# Set initial values

inits1 <- list(lambda20=0, lambda21=0, delta=structure(.Data=c(-0.5, -0.5, -0.5,

-0.5, -0.5, -0.5, -0.5, -0.5, -0.5, -0.5, -0.5, -0.5, -0.5, -0.5, -0.5,

-0.5, -0.5, -0.5, -0.5, -0.5, -0.5, -0.5, -0.5, -0.5, -0.5, -0.5), .Dim=c(13,2)), psi.2=0.25,

rho_w=0.25)

inits2 <- list(lambda20=50, lambda21=50, delta=structure(.Data=c(-5, -5,

-5, -5,-5, -5,-5, -5,-5, -5,-5, -5,-5, -5, -5, -5,

-5, -5,-5, -5,-5, -5,-5, -5,-5, -5), .Dim=c(13,2)),

psi.2=0.75, rho_w=0.75)

inits3 <- list(lambda20=10, lambda21=10, delta=structure(.Data=c(-50, -50,

-50, -50,-50, -50,-50, -50,-50, -50,-50, -50,-50, -50, -50, -50,

-50, -50,-50, -50,-50, -50,-50, -50,-50, -50), .Dim=c(13,2)),

psi.2=0.5, rho_w=0.5)

# Call WinBUGS and save results in variable out.re

out.re <- bugs(data, inits=list(inits1, inits2, inits3), model.file="code.txt",

parameters=c("lambda20", "lambda21", "psi2.sq"), n.thin=1,

n.chains=3, n.iter=150000, n.burnin=50000, bugs.directory=bd,

working.directory=work.dir, debug=FALSE)

# List all results as WinBUGS stats

print(out.re, digits=4)

output <- print(out.re, digits=4)

# Use coda to get output in usable format

coda.out <- read.bugs("coda1.txt")

# Make coda object into matrix

coda.m <- as.matrix(coda.out)

coda.m[1:10,]

# List all results as WinBUGS stats for each chain for lambda0

chain1lambda0 <- out.re$sims.array[,,"lambda20"][,1]

chain2lambda0 <- out.re$sims.array[,,"lambda20"][,2]

chain3lambda0 <- out.re$sims.array[,,"lambda20"][,3]

par(mfrow=c(1,1))

# History plots for lambda0 for different chains

plot(chain1lambda0, type="l", col="black", main="History Plot for Intercept", ylab="lambda0")

lines(chain2lambda0, type="l", col="blue")

lines(chain3lambda0, type="l", col="grey")

# Density plots for lambda0 for different chains

plot(density(chain1lambda0), lwd=3, col="black", main="Density Plot for Intercept", ylab="Density", xlim=c(-0.5, 0.7))

lines(density(chain2lambda0), lwd=3, col="blue")

lines(density(chain3lambda0), lwd=3, col="grey")

# Autocorrelation for all chains of lambda20

autocorr.plot(coda.m[,"lambda20"], lag.max=50, auto.layout=FALSE, main="Autocorrelation Plot for Intercept")

# List results as WinBUGS stats for each chain for lambda1

chain1lambda1 <- out.re$sims.array[,, "lambda21"][,1]

chain2lambda1 <- out.re$sims.array[,, "lambda21"][,2]

chain3lambda1 <- out.re$sims.array[,, "lambda21"][,3]

# History plot for different chains for lambda1

plot(chain1lambda1, type="l", col="black", main="History Plot for Slope", ylab="lambda1")

lines(chain2lambda1, type="l", col="blue")

lines(chain3lambda1, type="l", col="grey")

# Density plots for lambda1 for different chains

plot(density(chain1lambda1), lwd=3, col="black", main="Density Plot for Slope", ylab="Density", xlim=c(-1, 2.3))

lines(density(chain2lambda1), lwd=3, col="blue")

lines(density(chain3lambda1), lwd=3, col="grey")

# Autocorrelation plot for all chains of lambda1

autocorr.plot(coda.m[,"lambda21"], lag.max=50, auto.layout=FALSE, main="Autocorrelation Plot for Slope")

# List results as WinBUGS stats for each chain for conditional variance

chain1psisq <- out.re$sims.array[,, "psi2.sq"][,1]

chain2psisq <- out.re$sims.array[,, "psi2.sq"][,2]

chain3psisq <- out.re$sims.array[,, "psi2.sq"][,3]

# Plot these results for different chains

plot(chain1psisq, type="l", col="black", main="History Plot for Conditional Variance", ylab="Psi Squared")

lines(chain2psisq, type="l", col="blue")

lines(chain3psisq, type="l", col="grey")

# Density Plots for conditional variance with different chains

plot(density(chain1psisq), lwd=3, col="black", main="Density Plot for Conditional Variance", ylab="Density", xlim=c(0, 0.5), ylim=c(0, 100))

lines(density(chain2psisq), lwd=3, col="blue")

lines(density(chain3psisq), lwd=3, col="grey")

# Autocorrelation for all chains of conditional variance

autocorr.plot(coda.m[,"psi2.sq"], lag.max=50, auto.layout=FALSE, main="Autocorrelation Plot for Conditional Variance")

###################################################

# Cross Validation D&H Model for RCTs, cRWE and sRWE

###################################################

# Clear Environment

rm(list = ls())

# Set Working Directory

work.dir <- "…"

setwd(work.dir)

# Go to WinBUGS directory

bd <- "…"

# Load and check R2WinBUGS package

library(R2WinBUGS)

packageVersion("R2WinBUGS")

# Set number of iterations for MCMC iterations

n.iter <- 100000

n.burnin <- 50000

# Data on PFS and OS for RCTs

# Number of studies

num <- 13

# Authors of studies

author <- c("Guan 2011", "Hurwitz 2004", "Hecht 2011", "Hoff 2012",

"Tebbutt 2010", "Van Cutsem 2011", "Van Cutsem 2012",

"Hammerman 2014", "Lee 2017", "Devaux 2019", "Houts 2019",

"Dong 2015 vs Bendell 2012", "Yoshino 2007 vs Van Cutsem 2009")

# logHR for PFS

logHR_PFS <- c(-0.821, -0.616, -0.128, -0.174, -0.528, -0.186, -0.277,

-0.288, -0.329, -0.603, -0.202, -0.71, -0.622)

# se of logHR for PFS

logHR_PFS_se <- c(0.181, 0.09, 0.084, 0.075, 0.119, 0.077, 0.07, 0.0509,

0.0875, 0.299, 0.135, 0.119, 0.156)

# logHR for OS

logHR_OS <- c(-0.478, -0.416, 0.077, -0.062, -0.06, 0, -0.202, -0.288,

-0.248, -0.435, -0.129, -0.4, -0.414)

# se of logHR for OS

logHR_OS_se <- c(0.214, 0.1, 0.071, 0.089, 0.129, 0.073, 0.072, 0.0539,

0.0969, 0.307, 0.148, 0.133, 0.177)

# Effects on OS (log HR and corresponding CIs) for comparison with predicted effects

fin <- logHR_OS

fin.lci <- logHR_OS-1.96*logHR_OS_se

fin.uci <- logHR_OS+1.96*logHR_OS_se

# Effects on OS as hazard ratio (no log) and corresponding CIs

e.fin <- exp(logHR_OS)

e.fin.lci <- exp(logHR_OS-1.96*logHR_OS_se)

e.fin.uci <- exp(logHR_OS+1.96*logHR_OS_se)

# Create text file for bugs code of D&H model

dh_model_pred<-"model{

# Prior on the missing SE in the validation study i=index

se[index,2]~dunif(0.0001,15)

# Predicted effect on the final outcome in the validation study

pred.delta2<-delta[index,2]

# Within study precision matrix

rho_w~dunif(0,0.999)

for (i in 1:num) {

Prec_w[i,1:2,1:2] <- inverse(sigma[i,1:2,1:2])

sigma[i,1,1]<-pow(se[i,1],2)

sigma[i,2,2]<-pow(se[i,2],2)

sigma[i,1,2]<-sqrt(sigma[i,1,1])*sqrt(sigma[i,2,2])*rho_w

sigma[i,2,1]<-sqrt(sigma[i,1,1])*sqrt(sigma[i,2,2])*rho_w

}

# Random effects model

for (i in 1:num) {

Y[i,1:2]~dmnorm(delta[i,1:2], Prec_w[i,1:2,1:2])

# Daniels and Hughes model for the between study part:

delta[i,1]~dnorm(0.0, 0.001)

delta[i,2]~dnorm(eta2[i],prec2)

eta2[i]<-lambda0+lambda1*delta[i,1]

}

lambda0~dnorm(0.0, 1.0E-3)

lambda1~dnorm(0.0, 1.0E-3)

psi.2~dunif(0,2)

psi2.sq<-pow(psi.2,2)

prec2<-1/psi2.sq

}"

writeLines(dh_model_pred,"model_dh.txt")

# Set new variables for results of cross validation

pred.delta2.dh <- pred.cri.dh <- bias.dh <- cil.dh <- ciu.dh <- sd.dh <- array(0,num)

for(i in 1:num){

index <- i

Y <- se <- array(0, dim=c(num,2))

# Data

Y[,1] <- logHR_PFS

Y[,2] <- logHR_OS

se[,1] <- logHR_PFS_se

se[,2] <- logHR_OS_se

# Set OS in validation study i to be missing to be predicted

Y[i,2] <- NA

se[i,2] <- NA

data=list(Y=Y, se=se, num=num, index=i)

# Set initial values for missing outcome (OS) in validation study

Y0<-se0<-structure(rep(NA,num*2),dim=c(num,2))

Y0[index,2]<- -0.5

se0[index,2]<- 0.2

inits=list(list(psi.2 = 0.25, lambda1 = 0.0, lambda0 = 0.0, rho_w=0.25,

delta = structure(.Data = c(rep(-0.5,num),rep(-0.5,num)), .Dim=c(num,2)), Y=Y0,se=se0))

# Monitor prediction

para=c("psi2.sq", "pred.delta2")

# Run D&H Model

fit.dh<-bugs(data=data, inits=inits, para=para,

model.file="model_dh.txt",

n.chains=1, n.burnin=n.burnin, n.iter=n.iter, n.thin=1,

DIC=F, debug=F, save.history =F,

bugs.directory = bd, working.directory = work.dir)

x <- fit.dh$sims.matrix[,grep("pred.delta2",colnames(fit.dh$sims.matrix))]

pred.delta2.dh[i]<-mean(x)

bias.dh[i]<-logHR_OS[i]-pred.delta2.dh[i]

sd.dh[i] <- fit.dh$summary[grep("pred.delta2",rownames(fit.dh$summary)),c(2)]

pred.cri.dh[i]<-sqrt(logHR_OS_se[i]^2+sd.dh[i]^2)*2.00*1.96

cil.dh[i] <- pred.delta2.dh[i]-pred.cri.dh[i]/2.0

ciu.dh[i] <- pred.delta2.dh[i]+pred.cri.dh[i]/2.0

}

e.pred.dh<-exp(pred.delta2.dh)

e.pred.dh.lci<-exp(cil.dh)

e.pred.dh.uci<-exp(ciu.dh)

e.predictions.brma.dh<-data.frame(author,e.fin,e.fin.lci,e.fin.uci,

e.pred.dh,e.pred.dh.lci,e.pred.dh.uci,

fin,fin.lci,fin.uci,pred.delta2.dh,cil.dh,ciu.dh)

write.csv(e.predictions.brma.dh, file = "epredictions.dh.ms.csv", row.names = FALSE)

##########################################################

# BRMA PNF Model for RCTs, cRWE and sRWE

##########################################################

# Clear Environment

rm(list = ls())

# Set working directory

work.dir <- "…"

setwd(work.dir)

# Load and check R2WinBUGS package

library(R2WinBUGS)

packageVersion("R2WinBUGS")

# Go to WinBUGS directory

bd <- "…"

# Create data for WinBUGS

data <- list(num=13, Y=structure(.Data=c(-0.821, -0.616, -0.128, -0.174, -0.528, -0.186, -0.277, -0.288, -0.329, -0.603, -0.202, -0.71, -0.622, -0.478, -0.416, 0.077, -0.062, -0.06, 0, -0.202, -0.288, -0.248, -0.435, -0.129, -0.4, -0.414), .Dim=c(13,2)), se=structure(.Data=c(0.181, 0.09, 0.084, 0.075, 0.119, 0.077, 0.07, 0.0509, 0.0875, 0.299, 0.135, 0.119, 0.156, 0.214, 0.1, 0.071, 0.089, 0.129, 0.073, 0.072, 0.0539, 0.0969, 0.307, 0.148, 0.133, 0.177), .Dim=c(13,2)))

bugs.data(data)

# Create text file for WinBUGS code

code <- "model{

rho_w ~ dunif(0, 0.999)

for (i in 1:num){

Prec_w[i, 1:2, 1:2] <- inverse(sigma[i, 1:2, 1:2])

sigma[i,1,1] <- pow(se[i,1],2)

sigma[i,2,2] <- pow(se[i,2],2)

sigma[i,1,2] <- sqrt(sigma[i,1,1])*sqrt(sigma[i,2,2])*rho_w

sigma[i,2,1] <- sqrt(sigma[i,1,1])*sqrt(sigma[i,2,2])*rho_w

}

for (i in 1:num){

Y[i, 1:2] ~ dmnorm(delta[i, 1:2], Prec_w[i, 1:2, 1:2])

# PNF for between studies model

delta[i,1] ~ dnorm(eta1, prec1)

delta[i,2] ~ dnorm(eta2[i], prec2)

eta2[i] <- lambda20+lambda21*delta[i,1]

}

eta1 ~ dnorm(0.0, 0.001)

lambda20 ~ dnorm(0.0, 1.0E-3)

tau1 ~ dunif(0,2)

tau2 ~ dunif(0,2)

rho ~ dunif(-0.999, 0.999)

tau1.sq <- pow(tau1, 2)

prec1 <- 1/tau1.sq

tau2.sq <- pow(tau2,2)

psi2.sq <- tau2.sq-pow(lambda21,2)*tau1.sq

prec2 <- 1/psi2.sq

lambda21 <- rho*tau2/tau1

d1 <- eta1

d2 <- lambda20+lambda21*eta1

R2 <- pow(rho,2)

}"

writeLines(code,"code.txt")

# Set initial values

inits1 <- list(rho=0.5, eta1=0.0, lambda20=0.0, rho_w=0.25,

delta=structure(.Data=c(-0.5, -0.5, -0.5, -0.5, -0.5, -0.5, -0.5,

-0.5, -0.5, -0.5, -0.5, -0.5, -0.5, -0.5, -0.5, -0.5, -0.5, -0.5,

-0.5, -0.5, -0.5, -0.5,-0.5, -0.5, -0.5, -0.5),

.Dim = c(13,2)), tau1=0.25, tau2=0.25)

inits2 <- list(rho=0.75, eta1=5, lambda20=5, rho_w=0.75,

delta=structure(.Data=c(5, 5, 5, 5,5, 5,5, 5,5, 5,5, 5, 5,

5, 5, 5, 5, 5,5, 5,5, 5,5, 5,5, 5),

.Dim = c(13,2)), tau1=0.75, tau2=0.75)

inits3 <- list(rho=0.25, eta1=50, lambda20=50, rho_w=0.55,

delta=structure(.Data=c(50, 50, 50, 50,50, 50,50, 50,50,

50,50, 50,50, 50, 50, 50, 50, 50,50, 50,50, 50,50, 50,50, 50),

.Dim = c(13,2)), tau1=0.5, tau2=0.5)

# Call WinBUGS and save results in variable out.re

out.re <- bugs(data, inits=list(inits1, inits2, inits3), model.file="code.txt",

parameters=c("lambda20", "lambda21", "psi2.sq", "R2", "d1", "d2",

"rho", "tau1", "tau2"), n.thin=1, n.chains=3, n.iter=150000,

n.burnin=50000, bugs.directory=bd, working.directory=work.dir,

debug=FALSE)

# List all results as WinBUGS stats

print(out.re, digits=4)

output <- print(out.re, digits=4)

# Use coda to get output in usable format

coda.out <- read.bugs("coda1.txt")

#plot(coda.out)

# Make coda object into matrix

coda.m <- as.matrix(coda.out)

coda.m[1:10,]

# List all results as WinBUGS stats for each chain for lambda0

chain1lambda0 <- out.re$sims.array[,,"lambda20"][,1]

chain2lambda0 <- out.re$sims.array[,,"lambda20"][,2]

chain3lambda0 <- out.re$sims.array[,,"lambda20"][,3]

par(mfrow=c(1,1))

# History plots for lambda0 for different chains

plot(chain1lambda0, type="l", col="black", main="History Plot for Intercept", ylab="lambda0")

lines(chain2lambda0, type="l", col="blue")

lines(chain3lambda0, type="l", col="grey")

# Density plots for lambda0 for different chains

plot(density(chain1lambda0), lwd=3, col="black", main="Density Plot for Intercept", ylab="Density", xlim=c(-0.5, 0.7), ylim=c(0, 4))

lines(density(chain2lambda0), lwd=3, col="blue")

lines(density(chain3lambda0), lwd=3, col="grey")

# Autocorrelation for all chains of lambda20

autocorr.plot(coda.m[,"lambda20"], lag.max=50, auto.layout=FALSE, main="Autocorrelation Plot for Intercept")

# List results as WinBUGS stats for each chain for lambda1

chain1lambda1 <- out.re$sims.array[,, "lambda21"][,1]

chain2lambda1 <- out.re$sims.array[,, "lambda21"][,2]

chain3lambda1 <- out.re$sims.array[,, "lambda21"][,3]

# History plot for different chains for lambda1

plot(chain1lambda1, type="l", col="black", main="History Plot for Slope", ylab="lambda1")

lines(chain2lambda1, type="l", col="blue")

lines(chain3lambda1, type="l", col="grey")

# Density plots for lambda1 for different chains

plot(density(chain1lambda1), lwd=3, col="black", main="Density Plot for Slope", ylab="Density", xlim=c(-1, 2.3), ylim=c(0, 1.3))

lines(density(chain2lambda1), lwd=3, col="blue")

lines(density(chain3lambda1), lwd=3, col="grey")

# Autocorrelation plot for all chains of lambda1

autocorr.plot(coda.m[,"lambda21"], lag.max=50, auto.layout=FALSE, main="Autocorrelation Plot for Slope")

# List results as WinBUGS stats for each chain for conditional variance

chain1psisq <- out.re$sims.array[,, "psi2.sq"][,1]

chain2psisq <- out.re$sims.array[,, "psi2.sq"][,2]

chain3psisq <- out.re$sims.array[,, "psi2.sq"][,3]

# Plot these results for different chains

plot(chain1psisq, type="l", col="black", main="History Plot for Conditional Variance", ylab="Psi Squared")

lines(chain2psisq, type="l", col="blue")

lines(chain3psisq, type="l", col="grey")

# Density Plots for conditional variance with different chains

plot(density(chain1psisq), lwd=3, col="black", main="Density Plot for Conditional Variance", ylab="Density", xlim=c(0, 0.5), ylim=c(0, 80))

lines(density(chain2psisq), lwd=3, col="blue")

lines(density(chain3psisq), lwd=3, col="grey")

# Autocorrelation for all chains of conditional variance

autocorr.plot(coda.m[,"psi2.sq"], lag.max=50, auto.layout=FALSE, main="Autocorrelation Plot for Conditional Variance")

# List results as WinBUGS stats for each chain for correlation

chain1rho <- out.re$sims.array[,, "rho"][,1]

chain2rho <- out.re$sims.array[,, "rho"][,2]

chain3rho <- out.re$sims.array[,, "rho"][,3]

# Plot these results for different chains (history plot here)

plot(chain1rho, type="l", col="black", main="History Plot for Correlation", ylab="Correlation")

lines(chain2rho, type="l", col="blue")

lines(chain3rho, type="l", col="grey")

# Density plots for correlation with three chains

plot(density(chain1rho), lwd=3, col="black", main="Density Plot for Correlation", ylab="Density", xlim=c(0, 1))

lines(density(chain2rho), lwd=3, col="blue")

lines(density(chain3rho), lwd=3, col="grey")

# Autocorrelation for all chains of correlation

autocorr.plot(coda.m[,"rho"], lag.max=50, auto.layout=FALSE, main="Autocorrelation Plot for Correlation")

# List results as WinBUGS stats for each chain for R2

chain1R2 <- out.re$sims.array[,, "R2"][,1]

chain2R2 <- out.re$sims.array[,, "R2"][,2]

chain3R2 <- out.re$sims.array[,, "R2"][,3]

# Plot these results for different chains (history plot here)

plot(chain1R2, type="l", col="black", main="History Plot for R-squared", ylab="R-squared")

lines(chain2R2, type="l", col="blue")

lines(chain3R2, type="l", col="grey")

# Density plots for correlation with three chains

plot(density(chain1R2), lwd=3, col="black", main="Density Plot for R-squared", ylab="Density", xlim=c(0, 1))

lines(density(chain2R2), lwd=3, col="blue")

lines(density(chain3R2), lwd=3, col="grey")

# Autocorrelation for all chains of correlation

autocorr.plot(coda.m[,"R2"], lag.max=50, auto.layout=FALSE, main="Autocorrelation Plot for R-squared")

#########################################################

# Cross Validation for BRMA PNF Model with RCTs, cRWE and sRWE

#########################################################

# Clear Environment

rm(list = ls())

# Set Working Directory

work.dir <- "…"

setwd(work.dir)

# Go to WinBUGS directory

bd <- "…"

# Load and check R2WinBUGS package

library(R2WinBUGS)

packageVersion("R2WinBUGS")

# Set number of iterations for MCMC iterations

n.iter <- 100000

n.burnin <- 50000

# Data on PFS and OS for RCTs

num <- 13

# Authors of studies

author <- c("Guan 2011", "Hurwitz 2004", "Hecht 2011", "Hoff 2012",

"Tebbutt 2010", "Van Cutsem 2011", "Van Cutsem 2012",

"Hammerman 2014", "Lee 2017", "Devaux 2019", "Houts 2019",

"Dong 2015 vs Bendell 2012", "Yoshino 2007 vs Van Cutsem 2009")

# logHR for PFS

logHR_PFS <- c(-0.821, -0.616, -0.128, -0.174, -0.528, -0.186, -0.277,

-0.288, -0.329, -0.603, -0.202, -0.71, -0.622)

# se of logHR for PFS

logHR_PFS_se <- c(0.181, 0.09, 0.084, 0.075, 0.119, 0.077, 0.07, 0.0509,

0.0875, 0.299, 0.135, 0.119, 0.156)

# logHR for OS

logHR_OS <- c(-0.478, -0.416, 0.077, -0.062, -0.06, 0, -0.202, -0.288,

-0.248, -0.435, -0.129, -0.4, -0.414)

# se of logHR for OS

logHR_OS_se <- c(0.214, 0.1, 0.071, 0.089, 0.129, 0.073, 0.072, 0.0539,

0.0969, 0.307, 0.148, 0.133, 0.177)

# Effects on OS (log HR and corresponding CIs) for comparison with predicted effects

fin <- logHR_OS

fin.lci <- logHR_OS-1.96*logHR_OS_se

fin.uci <- logHR_OS+1.96*logHR_OS_se

# Effects on OS as hazard ratio (no log) and corresponding CIs

e.fin <- exp(logHR_OS)

e.fin.lci <- exp(logHR_OS-1.96*logHR_OS_se)

e.fin.uci <- exp(logHR_OS+1.96*logHR_OS_se)

# Create text file for BUGS code of the BRMA PNF Model

pnf_model_pred<-"model{

# Prior on missing se in validation study

se[index,2]~dunif(0.0001,15)

# Predicted effect on OS in validation study

pred.delta2<-delta[index,2]

# Within study precision matrix

rho_w~dunif(0,0.999)

for (i in 1:num) {

Prec_w[i,1:2,1:2] <- inverse(sigma[i,1:2,1:2])

sigma[i,1,1]<-pow(se[i,1],2)

sigma[i,2,2]<-pow(se[i,2],2)

sigma[i,1,2]<-sqrt(sigma[i,1,1])*sqrt(sigma[i,2,2])*rho_w

sigma[i,2,1]<-sqrt(sigma[i,1,1])*sqrt(sigma[i,2,2])*rho_w

}

# Random effects model

for (i in 1:num) {

Y[i,1:2]~dmnorm(delta[i,1:2], Prec_w[i,1:2,1:2])

# PNF Model

delta[i,1]~dnorm(eta1,prec1)

delta[i,2]~dnorm(eta2[i],prec2)

eta2[i]<-lambda0+lambda1*delta[i,1]

}

eta1~dnorm(0.0, 0.001)

lambda0~dnorm(0.0, 1.0E-3)

tau1~dunif(0,2)

tau2~dunif(0,2)

rho~dunif(-0.999,0.999)

tau1.sq<-pow(tau1,2)

prec1<-1/tau1.sq

tau2.sq<-pow(tau2,2)

psi2.sq<-tau2.sq-pow(lambda1,2)*tau1.sq

prec2<-1/psi2.sq

lambda1<-rho*tau2/tau1

d1<-eta1

d2<-lambda0+lambda1*eta1

R2<-pow(rho,2)

}"

writeLines(pnf_model_pred,"model_pnf.txt")

# Cross validation code for BRMA PNF model

pred.delta2.pnf<-pred.cri.pnf<-bias.pnf<-cil.pnf<-ciu.pnf<-sd.pnf<-array(0,num)

for(i in 1:num){

index<-i

Y<-se<-array(0,dim=c(num,2))

# Data

Y[,1]<-logHR_PFS

Y[,2]<-logHR_OS

se[,1]<-logHR_PFS_se

se[,2]<-logHR_OS_se

# Set OS in validation study i to be missing to be predicted

Y[i,2]<-NA

se[i,2]<-NA

data=list(Y=Y, se=se, num=num, index=i)

# Set initial values for missing outcome (OS) in validation study

Y0<-se0<-structure(rep(NA,num*2),dim=c(num,2))

Y0[index,2]<- -0.5

se0[index,2]<- 0.2

inits=list(list(rho = 0.5, eta1=0.0, lambda0 = 0.0,

tau1 = 0.25, tau2=0.25, rho_w=0.25,

delta = structure(.Data = c(rep(-0.5,num),rep(-0.5,num)), .Dim=c(num,2)),

Y=Y0,se=se0))

# Monitor prediction

para=c("psi2.sq", "pred.delta2")

#run PNF

fit.pnf<-bugs(data=data, inits=inits, para=para,

model.file="model_pnf.txt",

n.chains=1, n.burnin=n.burnin, n.iter=n.iter, n.thin=1,

DIC=F, debug=F, save.history =F,

bugs.directory = bd, working.directory = work.dir)

x <- fit.pnf$sims.matrix[,grep("pred.delta2",colnames(fit.pnf$sims.matrix))]

pred.delta2.pnf[i]<-mean(x)

bias.pnf[i]<-logHR_OS[i]-pred.delta2.pnf[i]

sd.pnf[i] <- fit.pnf$summary[grep("pred.delta2",rownames(fit.pnf$summary)),c(2)]

pred.cri.pnf[i]<-sqrt(logHR_OS_se[i]^2+sd.pnf[i]^2)*2.00*1.96

cil.pnf[i] <- pred.delta2.pnf[i]-pred.cri.pnf[i]/2.0

ciu.pnf[i] <- pred.delta2.pnf[i]+pred.cri.pnf[i]/2.0

}

e.pred.pnf<-exp(pred.delta2.pnf)

e.pred.pnf.lci<-exp(cil.pnf)

e.pred.pnf.uci<-exp(ciu.pnf)

e.predictions.brma.pnf<-data.frame(author,e.fin,e.fin.lci,e.fin.uci,

e.pred.pnf,e.pred.pnf.lci,e.pred.pnf.uci,

fin,fin.lci,fin.uci,pred.delta2.pnf,cil.pnf,ciu.pnf)

write.csv(e.predictions.brma.pnf, file = "epredictions.pnf.ms.csv", row.names = FALSE)

##############################################################

# BRMA PNF Model with bias adjustment for cRWE and sRWE studies

##############################################################

# Clear Environment

rm(list = ls())

# Set working directory

work.dir <- "…"

setwd(work.dir)

# Load and check R2WinBUGS package

library(R2WinBUGS)

packageVersion("R2WinBUGS")

# Go to WinBUGS directory

bd <- "…"

# Create data for WinBUGS

data <- list(Y=structure(.Data=c(-0.821, -0.616, -0.128, -0.174,

-0.528, -0.186, -0.277, -0.288, -0.329, -0.603, -0.202, -0.71,

-0.622, -0.478, -0.416, 0.077, -0.062, -0.06, 0, -0.202,

-0.288, -0.248, -0.435, -0.129, -0.4, -0.414),

.Dim=c(13,2)), se=structure(.Data=c(0.181, 0.09, 0.084, 0.075,

0.119, 0.077, 0.07, 0.0509, 0.0875, 0.299, 0.135, 0.119,

0.156, 0.214, 0.1, 0.071, 0.089, 0.129, 0.073, 0.072, 0.0539,

0.0969, 0.307, 0.148, 0.133, 0.177), .Dim=c(13,2)))

bugs.data(data)

# Create text file for WinBUGS code

code <- "model{

rho_w ~ dunif(0, 0.999)

for (i in 1:13){

Prec_w[i, 1:2, 1:2] <- inverse(sigma[i, 1:2, 1:2])

sigma[i,1,1] <- pow(se[i,1],2)

sigma[i,2,2] <- pow(se[i,2],2)

sigma[i,1,2] <- sqrt(sigma[i,1,1])*sqrt(sigma[i,2,2])*rho_w

sigma[i,2,1] <- sqrt(sigma[i,1,1])*sqrt(sigma[i,2,2])*rho_w

}

# RCTs

for (i in 1:7){

Y[i, 1:2] ~ dmnorm(delta[i, 1:2], Prec_w[i, 1:2, 1:2])

# PNF for between studies model

delta[i,1] ~ dnorm(eta1, prec1)

delta[i,2] ~ dnorm(eta2[i], prec2)

eta2[i] <- lambda20+lambda21*delta[i,1]

}

# cRWE

for(i in 8:11){

Y[i, 1:2] ~ dmnorm(deltacRWE[i, 1:2], Prec_w[i, 1:2, 1:2])

for(j in 1:2){

deltacRWE[i,j] <- delta[i,j] + beta1[i,j]

}

# PNF for between studies model

delta[i,1] ~ dnorm(eta1, prec1)

delta[i,2] ~ dnorm(eta2[i], prec2)

eta2[i] <- lambda20 + lambda21*delta[i,1]

# Normal distribution on bias terms

beta1[i,1] ~ dnorm(mean_beta11, prec_beta11)

beta1[i,2] ~ dnorm(mean_beta12, prec_beta12)

}

# sRWE

for(i in 12:13){

Y[i, 1:2] ~ dmnorm(deltasRWE[i, 1:2], Prec_w[i, 1:2, 1:2])

for(j in 1:2){

deltasRWE[i,j] <- delta[i,j] + beta2[i,j]

}

# PNF for between studies model

delta[i,1] ~ dnorm(eta1, prec1)

delta[i,2] ~ dnorm(eta2[i], prec2)

eta2[i] <- lambda20 + lambda21*delta[i,1]

# Normal distribution on bias terms

beta2[i,1] ~ dnorm(mean_beta21, prec_beta21)

beta2[i,2] ~ dnorm(mean_beta22, prec_beta22)

}

# Prior Distributions

mean_beta11 ~ dnorm(0, 0.001)

prec_beta11 <- 1/tau_beta11_sq

tau_beta11_sq <- tau_beta11 * tau_beta11

tau_beta11 ~ dunif(0,2)

mean_beta12 ~ dnorm(0, 0.001)

prec_beta12 <- 1/tau_beta12_sq

tau_beta12_sq <- tau_beta12 * tau_beta12

tau_beta12 ~ dunif(0,2)

mean_beta21 ~ dnorm(0, 0.001)

prec_beta21 <- 1/tau_beta21_sq

tau_beta21_sq <- tau_beta21 * tau_beta21

tau_beta21 ~ dunif(0,2)

mean_beta22 ~ dnorm(0, 0.001)

prec_beta22 <- 1/tau_beta22_sq

tau_beta22_sq <- tau_beta22 * tau_beta22

tau_beta22 ~ dunif(0,2)

eta1 ~ dnorm(0.0, 0.001)

lambda20 ~ dnorm(0.0, 1.0E-3)

tau1 ~ dunif(0,2)

tau2 ~ dunif(0,2)

rho ~ dunif(-0.999, 0.999)

tau1.sq <- pow(tau1, 2)

prec1 <- 1/tau1.sq

tau2.sq <- pow(tau2,2)

psi2.sq <- tau2.sq-pow(lambda21,2)*tau1.sq

prec2 <- 1/psi2.sq

lambda21 <- rho*tau2/tau1

d1 <- eta1

d2 <- lambda20+lambda21*eta1

R2 <- pow(rho,2)

}"

writeLines(code,"code.txt")

# Set initial values

inits1 <- list(rho=0.5, eta1=0.0, lambda20=0.0, rho_w=0.25,

delta=structure(.Data=c(-0.5, -0.5, -0.5, -0.5, -0.5,

-0.5, -0.5, -0.5, -0.5, -0.5, -0.5, -0.5, -0.5, -0.5,

-0.5, -0.5, -0.5, -0.5, -0.5, -0.5, -0.5, -0.5,-0.5,

-0.5, -0.5, -0.5), .Dim=c(13,2)), tau1=0.25,

tau2=0.25, beta1=structure(.Data=c(NA, NA, NA, NA, NA, NA,

NA, 0, 0, 0, 0, NA, NA, NA, NA, NA, NA, NA, 0, 0, 0, 0),

.Dim=c(11,2)), beta2=structure(.Data=c(NA, NA, NA, NA, NA,

NA, NA, NA, NA, NA, NA, 0, 0, NA, NA, NA, NA, NA, NA,

NA, NA, NA, NA, NA, 0, 0), .Dim=c(13,2)), mean_beta11=0,

mean_beta12=0, mean_beta21=0, mean_beta22=0, tau_beta11=0.5,

tau_beta12=0.5, tau_beta21=0.5, tau_beta22=0.5)

# Call WinBUGS and save results in variable out.re

out.re <- bugs(data, inits=list(inits1), model.file="code.txt",

parameters=c("lambda20", "lambda21", "psi2.sq", "R2", "d1", "d2",

"rho", "tau1", "tau2", "mean_beta11", "mean_beta12",

"mean_beta21", "mean_beta22", "tau_beta11", "tau_beta12",

"tau_beta21", "tau_beta22"), n.thin=1, n.chains=1,

n.iter=150000, n.burnin=50000, bugs.directory=bd,

working.directory=work.dir, debug=FALSE)

# List all results as WinBUGS stats

print(out.re, digits=4)

######################################################################

# Cross validation for model with bias adjustment for cRWE and sRWE studies

######################################################################

# Clear Environment

rm(list = ls())

# Set Working Directory

work.dir <- "…"

setwd(work.dir)

# Go to WinBUGS directory

bd <- "…"

# Load and check R2WinBUGS package

library(R2WinBUGS)

packageVersion("R2WinBUGS")

# Set number of iterations for MCMC iterations

n.iter <- 100000

n.burnin <- 50000

# Data on PFS and OS for RCTs

# Number of studies

num <- 13

# Authors of studies

author <- c("Guan 2011", "Hurwitz 2004", "Hecht 2011", "Hoff 2012",

"Tebbutt 2010", "Van Cutsem 2011", "Van Cutsem 2012",

"Hammerman 2014", "Lee 2017", "Devaux 2019", "Houts 2019",

"Dong 2015 vs Bendell 2012", "Yoshino 2007 vs Van Cutsem 2009")

# logHR for PFS

logHR_PFS <- c(-0.821, -0.616, -0.128, -0.174, -0.528, -0.186, -0.277,

-0.288, -0.329, -0.603, -0.202, -0.71, -0.622)

# se of logHR for PFS

logHR_PFS_se <- c(0.181, 0.09, 0.084, 0.075, 0.119, 0.077, 0.07, 0.0509,

0.0875, 0.299, 0.135, 0.119,0.156)

# logHR for OS

logHR_OS <- c(-0.478, -0.416, 0.077, -0.062, -0.06, 0, -0.202, -0.288,

-0.248, -0.435, -0.129, -0.4, -0.414)

# se of logHR for OS

logHR_OS_se <- c(0.214, 0.1, 0.071, 0.089, 0.129, 0.073, 0.072, 0.0539,

0.0969, 0.307, 0.148, 0.133, 0.177)

# Effects on OS (log HR and corresponding CIs) for comparison with predicted effects

fin <- logHR_OS

fin.lci <- logHR_OS-1.96*logHR_OS_se

fin.uci <- logHR_OS+1.96*logHR_OS_se

# Effects on OS as hazard ratio (no log) and corresponding CIs

e.fin <- exp(logHR_OS)

e.fin.lci <- exp(logHR_OS-1.96*logHR_OS_se)

e.fin.uci <- exp(logHR_OS+1.96*logHR_OS_se)

# Create text file for BUGS code of BRMA PNF model with bias adjustment

pnf_model_pred <- "model{

# Prior on missing se in validation study

se[index,2]~dunif(0.0001,15)

# Predicted effect on OS in validation study

pred.delta2<-delta[index,2]

# Within study precision matrix

rho_w~dunif(0,0.999)

for (i in 1:num) {

Prec_w[i,1:2,1:2] <- inverse(sigma[i,1:2,1:2])

sigma[i,1,1]<-pow(se[i,1],2)

sigma[i,2,2]<-pow(se[i,2],2)

sigma[i,1,2]<-sqrt(sigma[i,1,1])*sqrt(sigma[i,2,2])*rho_w

sigma[i,2,1]<-sqrt(sigma[i,1,1])*sqrt(sigma[i,2,2])*rho_w

}

# RCTs

for(i in 1:7){

Y[i, 1:2] ~ dmnorm(delta[i, 1:2], Prec_w[i, 1:2, 1:2])

# PNF for between studies model

delta[i,1] ~ dnorm(eta1, prec1)

delta[i,2] ~ dnorm(eta2[i], prec2)

eta2[i] <- lambda20+lambda21*delta[i,1]

}

# cRWE

for(i in 8:11){

Y[i, 1:2] ~ dmnorm(deltacRWE[i, 1:2], Prec_w[i, 1:2, 1:2])

for(j in 1:2){

deltacRWE[i,j] <- delta[i,j] + beta1[i,j]

}

# PNF for between studies model

delta[i,1] ~ dnorm(eta1, prec1)

delta[i,2] ~ dnorm(eta2[i], prec2)

eta2[i] <- lambda20 + lambda21*delta[i,1]

# Normal distribution on bias terms

beta1[i,1] ~ dnorm(mean_beta11, prec_beta11)

beta1[i,2] ~ dnorm(mean_beta12, prec_beta12)

}

# sRWE

for(i in 12:13){

Y[i, 1:2] ~ dmnorm(deltasRWE[i, 1:2], Prec_w[i, 1:2, 1:2])

for(j in 1:2){

deltasRWE[i,j] <- delta[i,j] + beta2[i,j]

}

# PNF for between studies model

delta[i,1] ~ dnorm(eta1, prec1)

delta[i,2] ~ dnorm(eta2[i], prec2)

eta2[i] <- lambda20 + lambda21*delta[i,1]

# Normal distribution on bias terms

beta2[i,1] ~ dnorm(mean_beta21, prec_beta21)

beta2[i,2] ~ dnorm(mean_beta22, prec_beta22)

}

# Prior Distributions

mean_beta11 ~ dnorm(0, 0.001)

prec_beta11 <- 1/tau_beta11_sq

tau_beta11_sq <- tau_beta11 * tau_beta11

tau_beta11 ~ dunif(0,2)

mean_beta12 ~ dnorm(0, 0.001)

prec_beta12 <- 1/tau_beta12_sq

tau_beta12_sq <- tau_beta12 * tau_beta12

tau_beta12 ~ dunif(0,2)

mean_beta21 ~ dnorm(0, 0.001)

prec_beta21 <- 1/tau_beta21_sq

tau_beta21_sq <- tau_beta21 * tau_beta21

tau_beta21 ~ dunif(0,2)

mean_beta22 ~ dnorm(0, 0.001)

prec_beta22 <- 1/tau_beta22_sq

tau_beta22_sq <- tau_beta22 * tau_beta22

tau_beta22 ~ dunif(0,2)

eta1 ~ dnorm(0.0, 0.001)

lambda20 ~ dnorm(0.0, 1.0E-3)

tau1 ~ dunif(0,2)

tau2 ~ dunif(0,2)

rho ~ dunif(-0.999, 0.999)

tau1.sq <- pow(tau1, 2)

prec1 <- 1/tau1.sq

tau2.sq <- pow(tau2,2)

psi2.sq <- tau2.sq-pow(lambda21,2)*tau1.sq

prec2 <- 1/psi2.sq

lambda21 <- rho*tau2/tau1

d1 <- eta1

d2 <- lambda20+lambda21*eta1

R2 <- pow(rho,2)

}"

writeLines(pnf_model_pred,"model_pnf.txt")

# Cross validation code for BRMA PNF model

pred.delta2.pnf<-pred.cri.pnf<-bias.pnf<-cil.pnf<-ciu.pnf<-sd.pnf<-array(0,num)

for(i in 1:num){

index<-i

Y<-se<-array(0,dim=c(num,2))

# Data

Y[,1]<-logHR_PFS

Y[,2]<-logHR_OS

se[,1]<-logHR_PFS_se

se[,2]<-logHR_OS_se

# Set OS in validation study i to be missing to be predicted

Y[i,2]<-NA

se[i,2]<-NA

data=list(Y=Y, se=se, num=num, index=i)

# Set initial values for missing outcome (OS) in validation study

Y0<-se0<-structure(rep(NA,num*2),dim=c(num,2))

Y0[index,2]<- -0.5

se0[index,2]<- 0.2

inits=list(list(rho=0.5, eta1=0.0, lambda20=0.0, rho_w=0.25,

delta=structure(.Data=c(-0.5, -0.5, -0.5, -0.5, -0.5,

-0.5, -0.5, -0.5, -0.5, -0.5, -0.5, -0.5, -0.5, -0.5,

-0.5, -0.5, -0.5, -0.5, -0.5, -0.5, -0.5, -0.5,-0.5,

-0.5, -0.5, -0.5), .Dim=c(13,2)), tau1=0.25,

tau2=0.25, beta1=structure(.Data=c(NA, NA, NA, NA, NA, NA,

NA, 0, 0, 0, 0, NA, NA, NA, NA, NA, NA, NA, 0, 0, 0, 0),

.Dim=c(11,2)), beta2=structure(.Data=c(NA, NA, NA, NA, NA,

NA, NA, NA, NA, NA, NA, 0, 0, NA, NA, NA, NA, NA, NA,

NA, NA, NA, NA, NA, 0, 0), .Dim=c(13,2)), mean_beta11=0,

mean_beta12=0, mean_beta21=0, mean_beta22=0, tau_beta11=0.5,

tau_beta12=0.5, tau_beta21=0.5, tau_beta22=0.5))

# Monitor prediction

para=c("psi2.sq", "pred.delta2")

#run PNF

fit.pnf<-bugs(data=data, inits=inits, para=para,

model.file="model_pnf.txt",

n.chains=1, n.burnin=n.burnin, n.iter=n.iter, n.thin=1,

DIC=F, debug=F, save.history =F,

bugs.directory = bd, working.directory = work.dir)

x <- fit.pnf$sims.matrix[,grep("pred.delta2",colnames(fit.pnf$sims.matrix))]

pred.delta2.pnf[i]<-mean(x)

bias.pnf[i]<-logHR_OS[i]-pred.delta2.pnf[i]

sd.pnf[i] <- fit.pnf$summary[grep("pred.delta2",rownames(fit.pnf$summary)),c(2)]

pred.cri.pnf[i]<-sqrt(logHR_OS_se[i]^2+sd.pnf[i]^2)*2.00*1.96

cil.pnf[i] <- pred.delta2.pnf[i]-pred.cri.pnf[i]/2.0

ciu.pnf[i] <- pred.delta2.pnf[i]+pred.cri.pnf[i]/2.0

}

e.pred.pnf<-exp(pred.delta2.pnf)

e.pred.pnf.lci<-exp(cil.pnf)

e.pred.pnf.uci<-exp(ciu.pnf)

e.predictions.brma.pnf<-data.frame(author,e.fin,e.fin.lci,e.fin.uci,

e.pred.pnf,e.pred.pnf.lci,e.pred.pnf.uci,

fin,fin.lci,fin.uci,pred.delta2.pnf,cil.pnf,ciu.pnf)

write.csv(e.predictions.brma.pnf, file = "epredictions.pnf.ms.csv", row.names = FALSE)

**Appendix I: History Plots**


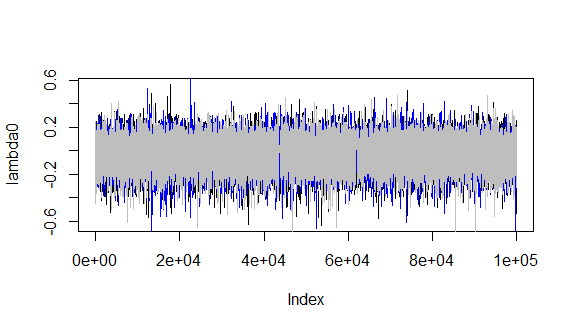


Figure I1: History plot for $\lambda_{0}$ in D&H model.


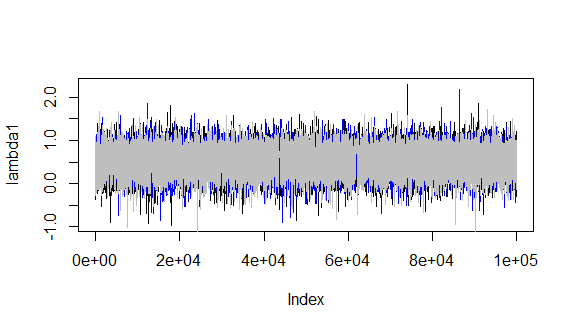


Figure I2: History plot for $\lambda_{1}$ in D&H model.


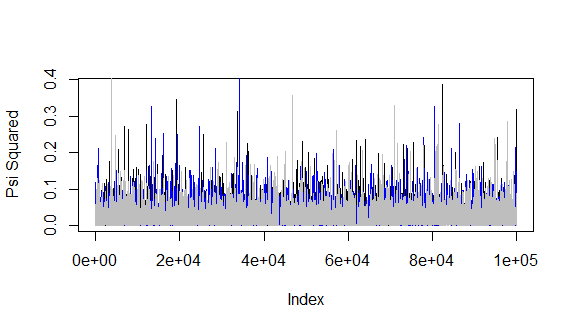


Figure I3: History Plot for $\psi_{2}^{2}$ in D&H model.

**Appendix J: Results of D&H and BRMA PNF models using alternative within-study correlation**

Table J1: Surrogacy criteria obtained from D&H model applied to data from RCTs, comparative RWE (cRWE) and matched single arm RWE (sRWE) using within-study correlation of 0.52 obtained from bootstrapping.

|  | RCTs | RCTs & cRWE | RCTs, cRWE & sRWE |
| --- | --- | --- | --- |
| $\lambda_{0}$ | 0.11 (-0.12, 0.33) | 0.052 (-0.13, 0.23) | 0.049 (-0.10, 0.20) |
| $\lambda_{1}$ | 0.72 (0.14, 1.28) | 0.70 (0.20, 1.19) | 0.69 (0.31, 1.05) |
| $\psi_{2}^{2}$ | 0.0077 (0.0000, 0.10) | 0.0094 (0.0004, 0.050) | 0.0075 (0.0005, 0.035) |
| Absolute discrepancy, Median (Range) | 0.097 (0.00071, 0.27) | 0.078 (0.0077, 0.31) | 0.069 (0.0097, 0.28) |
| $w_{\hat{Y}_{2j}}/w_{Y_{2j}}$, Median (Range) | 2.77 (1.71, 3.61) | 2.05 (1.33, 2.37) | 1.71 (1.28, 2.09) |

Table J2: Surrogacy criteria obtained from BRMA PNF model applied to data from RCTs, comparative RWE (cRWE) and matched single arm RWE (sRWE) using within-study correlation of 0.52 obtained from bootstrapping.

|  | RCTs | RCTs & cRWE | RCTs, cRWE & sRWE | Bias Adjusted RCTs, cRWE & sRWE |
| --- | --- | --- | --- | --- |
| $d_{1}$ | -0.36  (-0.61, -0.13) | -0.34  (-0.48, -0.21) | -0.39  (-0.53, -0.25) | -0.36  (-0.55, -0.18) |
| $d_{2}$ | -0.14  (-0.35, 0.045) | -0.18  (-0.31, -0.055) | -0.21  (-0.34, -0.10) | -0.14  (-0.30, 0.0038) |
| $\rho$ | 0.77  (-0.20, 0.99) | 0.70  (-0.20, 0.97) | 0.79  (0.14, 0.98) | 0.78  (-0.089, 0.99) |
| $\tau_{1}$ | 0.24  (0.11, 0.59) | 0.17  (0.070, 0.35) | 0.20  (0.11, 0.36) | 0.19  (0.079, 0.40) |
| $\tau_{2}$ | 0.19  (0.074, 0.47) | 0.16  (0.075, 0.32) | 0.16  (0.083, 0.30) | 0.15  (0.053, 0.32) |
| $\lambda_{0}$ | 0.060  (-0.21, 0.34) | 0.018  (-0.24, 0.29) | 0.024  (-0.17, 0.23) | 0.061  (-0.16, 0.29) |
| $\lambda_{1}$ | 0.57  (-0.13, 1.33) | 0.58  (-0.17, 1.42) | 0.61  (0.093, 1.15) | 0.57  (-0.052, 1.24) |
| $\psi_{2}^{2}$ | 0.012  (0.0008, 0.099) | 0.011  (0.0015, 0.047) | 0.0090  (0.0014, 0.037) | 0.0075  (0.0003, 0.048) |
| $R^{2}$ | 0.59  (0.0047, 0.98) | 0.49  (0.0046, 0.94) | 0.62  (0.029, 0.95) | 0.60  (0.0073, 0.98) |
| $\alpha_{1}$ |  |  |  | 0.031  (-0.41, 0.47) |
| $\alpha_{2}$ |  |  |  | -0.12 (-0.49, 0.27) |
| $\beta_{1}$ |  |  |  | -0.31  (-1.66, 1.06) |
| $\beta_{2}$ |  |  |  | -0.26  (-1.60, 1.07) |
| Absolute Discrepancy, Median (Range) | 0.15  (0.016, 0.25) | 0.16  (0.0060, 0.24) | 0.12  (0.0042, 0.25) | 0.16  (0.014, 0.28) |
| $w_{\hat{Y}_{2j}}/w_{Y_{2j}}$, Median (Range) | 2.70  (1.57, 3.15) | 2.02  (1.15, 2.43) | 1.70  (1.14, 2.23) | 1.83  (1.19, 3.45) |

**Appendix K: Results of D&H and BRMA PNF models using Unif(0,100) for SD**

Table K1: Surrogacy criteria from D&H model applied to data from RCTs, comparative RWE (cRWE) and matched single arm RWE (sRWE) using Unif(0,100) for prior distribution on standard deviation

|  | RCTs | RCTs & cRWE | RCTs, cRWE & sRWE |
| --- | --- | --- | --- |
| $\lambda_{0}$ | 0.10 (-0.13, 0.34) | 0.051 (-0.13, 0.23) | 0.050 (-0.10, 0.20) |
| $\lambda_{1}$ | 0.71 (0.12, 1.29) | 0.69 (0.20, 1.17) | 0.69 (0.31, 1.04) |
| $\psi_{2}^{2}$ | 0.0089 (0.0000, 0.11) | 0.010 (0.0002, 0.051) | 0.0088 (0.0004, 0.037) |
| Absolute discrepancy, Median (Range) | 0.092 (0.0068, 0.27) | 0.070 (0.0079, 0.30) | 0.072 (0.0074, 0.28) |
| $w_{\hat{Y}_{2j}}/w_{Y_{2j}}$, Median (Range) | 2.99 (1.71, 3.74) | 2.09 (1.33, 2.40) | 1.72 (1.28, 2.15) |

Table K2: Surrogacy criteria from BRMA PNF model applied to data from RCTs, comparative RWE (cRWE) and matched single arm RWE (sRWE) using Unif(0,100) for prior distribution on between-studies heterogeneity parameters

|  | RCTs | RCTs & cRWE | RCTs, cRWE & sRWE | RCTs, cRWE & sRWE Bias Adjusted |
| --- | --- | --- | --- | --- |
| $d_{1}$ | -0.36  (-0.61, -0.13) | -0.34  (-0.49, -0.21) | -0.39  (-0.53, -0.26) | -0.36  (-0.55, -0.18) |
| $d_{2}$ | -0.14  (-0.35, 0.046) | -0.18  (-0.31, -0.052) | -0.21  (-0.33, -0.093) | -0.14  (-0.30, 0.0075) |
| $\rho$ | 0.75  (-0.22, 0.99) | 0.66  (-0.30, 0.97) | 0.74  (0.065, 0.97) | 0.73  (-0.22, 0.98) |
| $\tau_{1}$ | 0.25  (0.11, 0.59) | 0.18  (0.079, 0.36) | 0.20  (0.11, 0.36) | 0.19  (0.091, 0.39) |
| $\tau_{2}$ | 0.19  (0.074, 0.47) | 0.15  (0.072, 0.31) | 0.16  (0.080, 0.30) | 0.15  (0.054, 0.32) |
| $\lambda_{0}$ | 0.054  (-0.22, 0.34) | 0.0068  (-0.26, 0.28) | 0.012  (-0.19, 0.21) | 0.048  (-0.19, 0.29) |
| $\lambda_{1}$ | 0.55  (-0.15, 1.33) | 0.54  (-0.25, 1.40) | 0.56  (0.041, 1.12) | 0.52  (-0.14, 1.23) |
| $\psi_{2}^{2}$ | 0.013  (0.0008, 0.10) | 0.011  (0.0014, 0.048) | 0.0099  (0.0018, 0.037) | 0.0084  (0.0005, 0.050) |
| $R^{2}$ | 0.57  (0.0043, 0.98) | 0.43  (0.0023, 0.94) | 0.55  (0.014, 0.94) | 0.54  (0.0046, 0.97) |
| $\alpha_{1}$ |  |  |  | 0.036  (-0.40, 0.47) |
| $\alpha_{2}$ |  |  |  | -0.12  (-0.49, 0.27) |
| $\beta_{1}$ |  |  |  | -0.31  (-1.68, 1.07) |
| $\beta_{2}$ |  |  |  | -0.27  (-1.60, 1.04) |
| Absolute Discrepancy, Median (Range) | 0.16  (0.018, 0.26) | 0.16  (0.0046, 0.23) | 0.13  (0.0036, 0.24) | 0.16  (0.011, 0.28) |
| $w_{\hat{Y}_{2j}}/w_{Y_{2j}}$, Median (Range) | 2.80  (1.60, 3.39) | 2.09  (1.15, 2.49) | 1.72  (1.14, 2.28) | 1.80  (1.17, 3.50) |

**Appendix L: Bubble Plot for BRMA PNF**


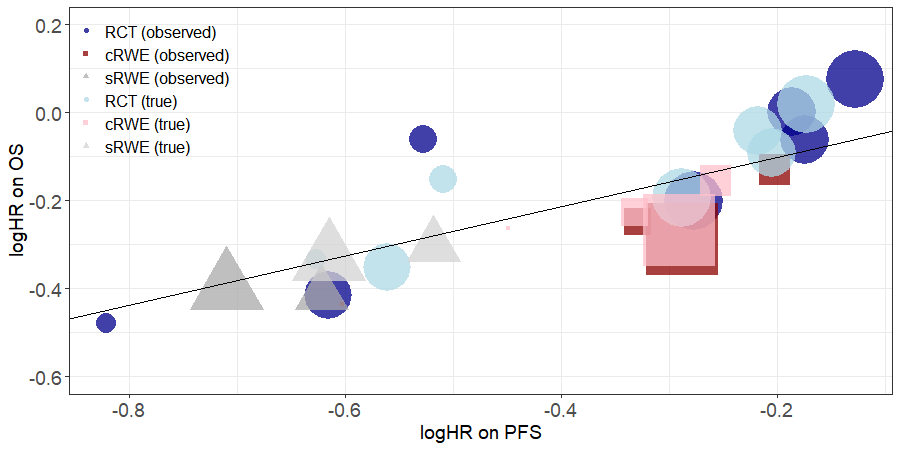


Figure L1: Scatterplot of logHRs on OS against logHRs on PFS. Dark blue circles, dark red squares and dark grey triangles show the observed treatment effects from RCTs, cRWE and matched sRWE respectively. Light blue circles, light red squares and light grey triangles show true treatment effects estimated by BRMA model from RCTs, cRWE and matched sRWE respectively. Black line shows the linear relationship between logHR on PFS and logHR on OS obtained from BRMA model conducted using all sources of evidence. Size of points refers to size of study
